# *In vivo* assembly and large-scale purification of a GPCR - Gα fusion with Gβγ, and characterization of the active complex

**DOI:** 10.1371/journal.pone.0210131

**Published:** 2019-01-08

**Authors:** Abhinav Kumar, Andreas Plückthun

**Affiliations:** Department of Biochemistry, University of Zürich, Zürich, Switzerland; Weizmann Institute of Science, ISRAEL

## Abstract

G protein coupled receptors (GPCRs) are central players in recognizing a variety of stimuli to mediate diverse cellular responses. This myriad of functions is accomplished by their modular interactions with downstream intracellular transducers, such as heterotrimeric G proteins and arrestins. Assembling a specific GPCR–G protein pair as a purified complex for their structural and functional investigations remains a challenging task, however, because of the low affinity of the interaction. Here, we optimized fusion constructs of the Gα subunit of the heterotrimeric G protein and engineered versions of rat Neurotensin receptor 1 (NTR1), coexpressed and assembled in vivo with Gβ and Gγ. This was achieved by using the baculovirus-based MultiBac system. We thus generated a functional receptor–G protein fusion complex, which can be efficiently purified using ligand-based affinity chromatography on large scales. Additionally, we utilized a purification method based on a designed ankyrin repeat protein tightly binding to Green Fluorescent Protein (GFP-DARPin) that may be used as a generic approach for a large-scale purification of GPCR–G protein fusion complexes for which no ligands column can be generated. The purification methods described herein will support future studies that aim to understand the structural and functional framework of GPCR activation and signaling.

## 1. Introduction

G protein-coupled receptors (GPCRs) are the largest group of membrane receptors controlling many aspects of physiology. They are the target of over 30% of the marketed pharmaceuticals [1]. Ligand binding causes GPCRs to engage with numerous downstream transducers, of which heterotrimeric G proteins and β-arrestins are well studied [1, 2]. A recent increase in the number of solved cryo-electron microscopy (cryo-EM) structures of some GPCRs in complex with different G protein subtypes is making it possible to understand the mechanism of activation and the structural basis of the GPCR–G protein selectivity [3–5]. However, many GPCRs and their complexes with other G protein subtypes (along with other transducers) remain to be explored. Thus, there is a need for a method that can be utilized for a rapid assembly and purification of GPCR–G protein complexes. Such methods will support the endeavors for understanding the structural basis of the plasticity observed in GPCRs and their complexes.

Neurotensin receptor 1 (NTR1) belongs to the prototypical class A GPCRs and mediates its function by coupling mainly to Gq-, and partially to Gi- and Gs-type G proteins. This adaptability makes this receptor particularly interesting for study. NTR1 has been implicated in various neurodegenerative disorders such as Parkinson’s disease and schizophrenia [6]. It is also involved in the progression of various types of cancers [7–9] and obesity [10]. Neurotensin (NT) is a tridecapeptide ligand of NTR1 that performs a dual function: it acts as a neuromodulator in the central nervous system (CNS) [11] and as a local hormone in the periphery [12]. The NTR1 mutants previously generated using directed-evolution-based methods show higher expression levels and better stability in detergents compared to the wild-type receptor [13–16]. Their enhanced biophysical properties acquired using directed-evolution methods paved the way for the structural characterization of three evolved variants of NTR1 [17]. Moreover, being able to couple to the G protein, the evolved mutants of NTR1 are also attractive targets for structural and functional studies of their complexes with G proteins [18]. However, the previously described method for the *in vitro* assembly of the NTR1 –G protein complex is comparatively laborious when scaled up [18].

The fusion of the receptor with the G protein alpha subunit, Gα, has long been investigated as a model system to study various aspects of GPCR and G protein functionality [19, 20]. Typically, such fusions are generated by a covalent assembly of the N-terminus of a G protein α subunit in frame with the C-terminal region of a GPCR. Over the years, the concept of receptor-Gα fusions has been utilized with several other GPCRs and Gα pairs [20], including the NTR1 [21]. The fusion is believed to ensure a 1:1 stoichiometry and a physical vicinity between the GPCR and the Gα subunit. This may explain the observed increase in the functional-coupling efficiency of the Gα subunit to the fused receptor [22]. Thus, even though the fusion strategy is inherently artificial, it has been proposed as a strategy for facilitating structural studies of receptor interactions [23]. Analogous to the receptor-Gα fusion, constructs comprising receptor– β-arrestin fusions have been utilized for structural characterization [24].

In the present study, we engineered a rat NTR1-Gα fusion with a minimal tether-length and devised a co-purification strategy with co-expressed Gβ and Gγ. We efficiently utilized this NTR1-Gα fusion construct for a preparative scale production of NTR1 –G protein signaling complex. The presented method uses the baculovirus-based MultiBac system [25] to co-express the receptor-Gα fusion along with the Gβ and the Gγ subunits, simultaneously. The expression method thus allows an *in vivo* assembly of the signaling complex. The NTR1 –Gαβγ fusion complex thus formed can then be efficiently purified using a cleavable ligand-affinity based column [26] in functional form resulting in a high purity. The effect of various buffer conditions and detergents were probed on the purified fusion complex, elucidating the range of conditions where protein remains functional and well folded.

Moreover, we also tested a purification method based on a designed ankyrin repeat protein binding to green fluorescent protein (GFP-DARPin) [27] for the purification of the NTR1 –G protein fusion complex. The GFP-DARPin-based purification method was equally efficient as compared to the ligand-affinity-based method. The GFP-DARPin-based purification is a generic method and thus an attractive alternative that may be applied to other GPCR–G protein complexes for which no agonist-affinity purification can be designed. The successful production for the signaling complex using the MultiBac approach and the generic purification strategy described herein provides an attractive and simple approach for a preparative-scale purification of other unexplored GPCR–G protein complex systems. A combination of both methods presented here may assist in future ventures for studying biophysical and structural properties of this pharmacologically important class of biomolecules.

## 2. Results and discussion

### 2.1 Optimization of the tether-length in NTR1-Gα fusion constructs

Ligand-regulated binding of [^35^S]GTPγS is one of the most widely used methods to measure the functional coupling of GPCR with a heterotrimeric G protein [28]. We used the mutant TM86V-L167R of NTR1 to optimize the fusion construct with this assay. The mutant TM86V has been obtained by directed evolution for enhanced functional expression and stability in short-chain detergents [13–16]. It has acquired several mutations, one of which was the introduction of Leu instead of Arg in the conserved E/DRY motif, which locks the receptor in the inactive form on the cytoplasmic side and thus stabilizes it significantly [17]. The reversion of this mutation, L167R, improved its functional coupling with the G protein, as detected by ligand-dependent [^35^S]GTPγS assay [18].

**Fig 1:**
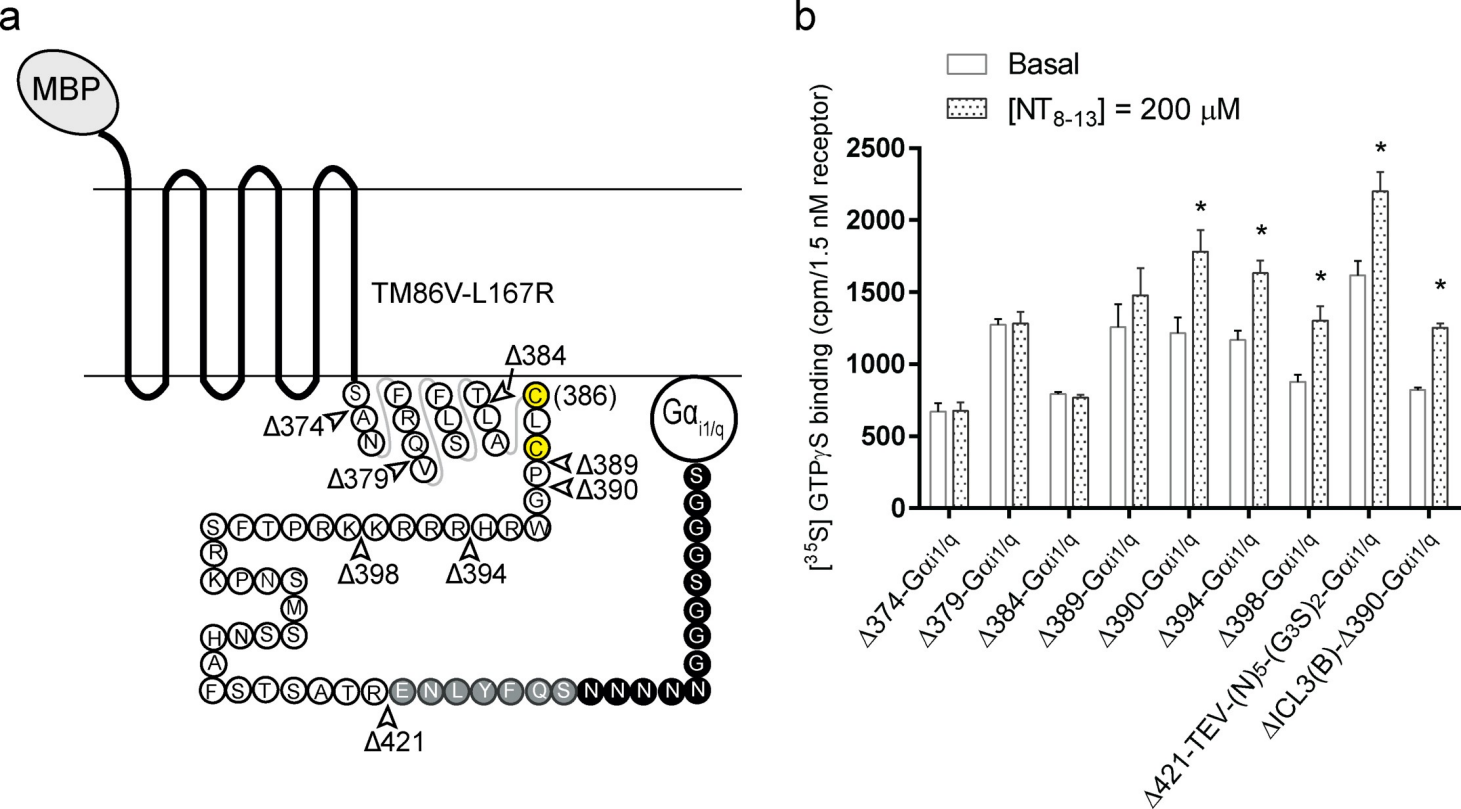
Ligand-induced [^35^S]GTPγS binding of different fusions of Gα_i1/q_ at the C-terminus of rat NTR1 mutant, TM86V-L167R. (**a**) Schematic diagram of the longest NTR-Gα fusion construct. Residues after helix 7 are shown in circles and helix 8 (from Ser373) is indicated as a schematic projection. Potential palmitoylation sites (Cys386 and Cys388) are shown in yellow circles. In the longest construct, the C-terminus of the receptor was fused to the N-terminus of the Gα_i1/q_ chimera with a TEV cleavage site (grey circles) and a (N_5_)-(G_3_S)_2_ linker (black circles) in between. In all the other shorter constructs, the Gα_i1/q_ was directly fused to the different C-terminal amino acid positions. The arrows (empty) indicate the location of truncation sites as evaluated in panel b and the number after “Δ” indicates the first missing residue. (**b**) NT_8-13_-induced [^35^S]GTPγS assay on *E*. *coli* membranes containing the MBP-TM86V-L167R-Gα_i1/q_ constructs. A complete or partial deletion of helix 8 in the fusion construct abrogates the signaling competency. Data are given as a mean ± s.e.m. of 2–7 independent experiments performed in at least in duplicates. *p<0.05 compared to the basal level calculated by Student’s *t*-test. The response (percentage increase in the binding of [^35^S]GTPγS above basal level) at 200 μM NT are 46.6±15% for Δ390-Gα_i1/q_ (n = 5); 39.8±9% for Δ394-Gα_i1/q_ (n = 7); 48.3±13% for Δ398-Gα_i1/q_ (n = 4); 36.1±10% for Δ421-TEV-(N_5_)-(G_3_S)_2_-Gα_i1/q_ (n = 7)).

Taking advantage of the high expression level of TM86V-L167R in a bacterial system and its ability to at least modestly couple to G protein, we created the fusion of TM86V-L167R with the Gα subunit. For these experiments, Gα_i1_ was preferred over Gα_q_, as it can be easily expressed in a bacterial system leading to a higher yield. Additionally, Gα_i1_ has a higher rate of basal nucleotide exchange compared to other Gα subunits, thus giving a better signal-to-background ratio in the [^35^S]GTPγS assay [28]. In order to impart a better coupling to NTR1, we exchanged the five C-terminal amino acids of Gα_i1_ to those of Gα_q_ [21]. The longest fusion construct was termed as Δ421-TEV-(N_5_)-(G_3_S)_2_-Gα_i1/q_, where 421 denotes the first missing amino acid of the GPCR (see Fig 1A, and Section 4.2 for details). For an efficient export to the bacterial inner-membrane, the *E*. *coli* maltose binding protein (MBP) including its signal sequence was fused N-terminally to all fusion constructs [29].

One can envision that the length of the tether between the receptor and the Gα subunit might influence the optimal orientation of the two proteins for efficient coupling. A very short linker between the two proteins might impair their coupling while a very long linker may allow many possible orientations and decrease the local concentration. Furthermore, the conformational flexibility imparted due to the many possible orientations is not favorable for structural characterizations either by X-ray crystallography or by cryo-EM [30]. Thus, we determined the shortest tether-length between the two proteins that still allowed an efficient agonist-induced binding of [^35^S]GTPγS. We generated various fusion constructs by systematically truncating the C-terminus of the receptor. We denote the first missing amino acid after “Δ” in the truncation constructs. Only the longest construct (Fig 1A) contains a TEV protease cleavage site followed by a (N_5_)-(G_3_S)_2_ linker before the Gα_i1/q_ domain. The next shorter construct and all the others are direct fusions of the respective C-terminus of the receptor to the N-terminus of the Gα_i1/q_ domain. The designed fusion constructs were individually expressed using the bacterial expression system. The truncated fusion constructs were then evaluated for their ability to allow functional Gα coupling using the agonist-induced [^35^S]GTPγS assay in bacterial membranes. The optimal short-tether construct thus found was subsequently used in the baculovirus-based MultiBac system to generate a functional NTR1 –G protein complex (Section 2.2).

The [^35^S]GTPγS assays of the selected fusion constructs showed that a complete deletion of helix 8 (after Ser-373), i.e. construct Δ374-Gα_i1/q_, totally impaired the ability of agonist-induced coupling of Gα_i1/q_ with the receptor (Fig 1B). The effect was also observed in the constructs where helix 8 was only partially deleted, Δ379-Gα_i1/q_ and Δ384-Gα_i1/q_. The inability of these constructs to trigger agonist-induced [^35^S]GTPγS binding supports the important functional aspect of helix 8 in a GPCR for coupling with a G protein, and has been studied using other unfused GPCRs [31–34]. Fusing the Gα_i1/q_ immediately after the potential palmitoylation site [35] Cys388, i.e. construct Δ389-Gα_i1/q_, also did not result in statistically significant amplitude of response (percentage increase in binding of [^35^S]GTPγS compared to the basal level) measured at 200 μM NT_8-13_. However, a statistically significant response was observed in Δ390-Gα_i1/q_, and this construct was the most consistent in showing a higher degree of response compared to the other constructs (overall, 46.6±15%, n = 5) (Fig 1B). Any further lengthening of the C-terminal truncation did not have a major effect on the extent of the maximal response (Fig 1B).

The intracellular loop 3 (ICL3) of the rat NTR1 contains several basic residues, which seem to be cleaved by *E*. *coli* proteases, and the sequence is also similar to that of the recognition sequence of HRV 3C protease. The deletions in the ICL3 were necessary to obtain a homogeneous preparation and for subsequent crystallization of the receptor alone [17, 26]. For this purpose, amino acids Glu273 to Thr290 of the construct Δ390-Gα_i1/q_ were removed, and indeed the construct termed ΔICL3(B)-Δ390-Gα_i1/q_ showed a slightly improved agonist-based coupling in the [^35^S]GTPγS assay, 52.3±4% (n = 3). A marginally improved receptor-coupling associated with the ICL3 deletion construct is consistent with the observations made using a GDP/[^35^S]GTPγS exchange assay performed previously with TM86V-L167R and TM86V-ΔICL3 L167R, using unfused receptor mutant and independently expressed, purified heterotrimeric G protein [17, 18].

It is worth noting that there is no correlation between the tether length and the maximal response. Specifically, the maximal response of the longest construct (Δ421-TEV-(N_5_)-(G_3_S)_2_-Gα_i1/q_) was not higher, but rather lower than that of the other shorter constructs (Δ390-Gα_i1/q_ or Δ398-Gα_i1/q_). It is possible that with excessive tether length, a very high mobility of the Gα_i1/q_ relative to the receptor was obtained, and thus a relatively lower local concentration, which might have led to a reduced probability of engagement with the receptor. The observation that a reduced tether length between receptor and Gα_i1/q_ improves the coupling efficiency as compared to when Gα_i1/q_ is fused at the far C-terminus (with additional linkers) of the receptor, observed here with TM86V-L167R, is consistent with a similar observation made using a neurokinin receptor (NK1)-Gα_q_ fusion protein [36].

Thus, using a gradual truncation of the C-terminal tail of the receptor in the fusion constructs and subsequent [^35^S]GTPγS assays, we were able to find a construct with optimal short tether-length that showed agonist-induced receptor-coupling. These experiments were carried out in the context of the stabilized receptor TM86V-L167R carrying no loop deletions. However, for the purification of the fusion complex, construct ΔICL3(B)-Δ390-Gα_i1/q_ was preferred, as it had a truncated ICL3 and thus HRV-3C-mediated proteolysis could be avoided (as mentioned earlier). In the next step, we utilized the designed optimally short fusion construct for producing milligram quantities of purified NTR1-G protein complex.

### 2.2 MultiBac assembly of the GPCR–G protein complex

The MultiBac system is a baculovirus expression system specially designed for the production of eukaryotic multi-protein complexes with several subunits [25, 37]. The method uses multigene cassettes generated by a site-specific Cre-Lox recombination of specialized plasmids that are called ‘acceptors’ (pFL) and ‘donors’ (pIDC). The recombined plasmid then integrates into an engineered MultiBac genome using a Tn7 transposition site. The engineered MultiBac genome also encodes an enhanced yellow fluorescent protein (eYFP), which allows an easy tracking and monitoring of virus performance and expression [38].

As the acceptor (pFL) vector permits a direct integration into the viral genome, thus allowing a relatively quick monitoring of expression, the receptor-Gα gene construct was cloned into the acceptor (pFL) vector instead of donor vectors (pIDC). Unlike in the bacterial expression system, where MBP with its signal sequence was used to transport the fusion protein to the inner membrane [29], a mellitin secretion signal sequence was placed upstream of the receptor for expression in insect cells. In parallel, the Gβ and the Gγ subunits were cloned into two independent donor (pIDC) vectors. The choice of the subtype of Gβ and Gγ was based on previous findings that Gβ_1_ and Gγ_1_ (along with Gγ_11_) in the context of Gα_i1_ show the best interaction both with wild-type NTR1 and the evolved NTR1 variants [18].

**Fig 2:**
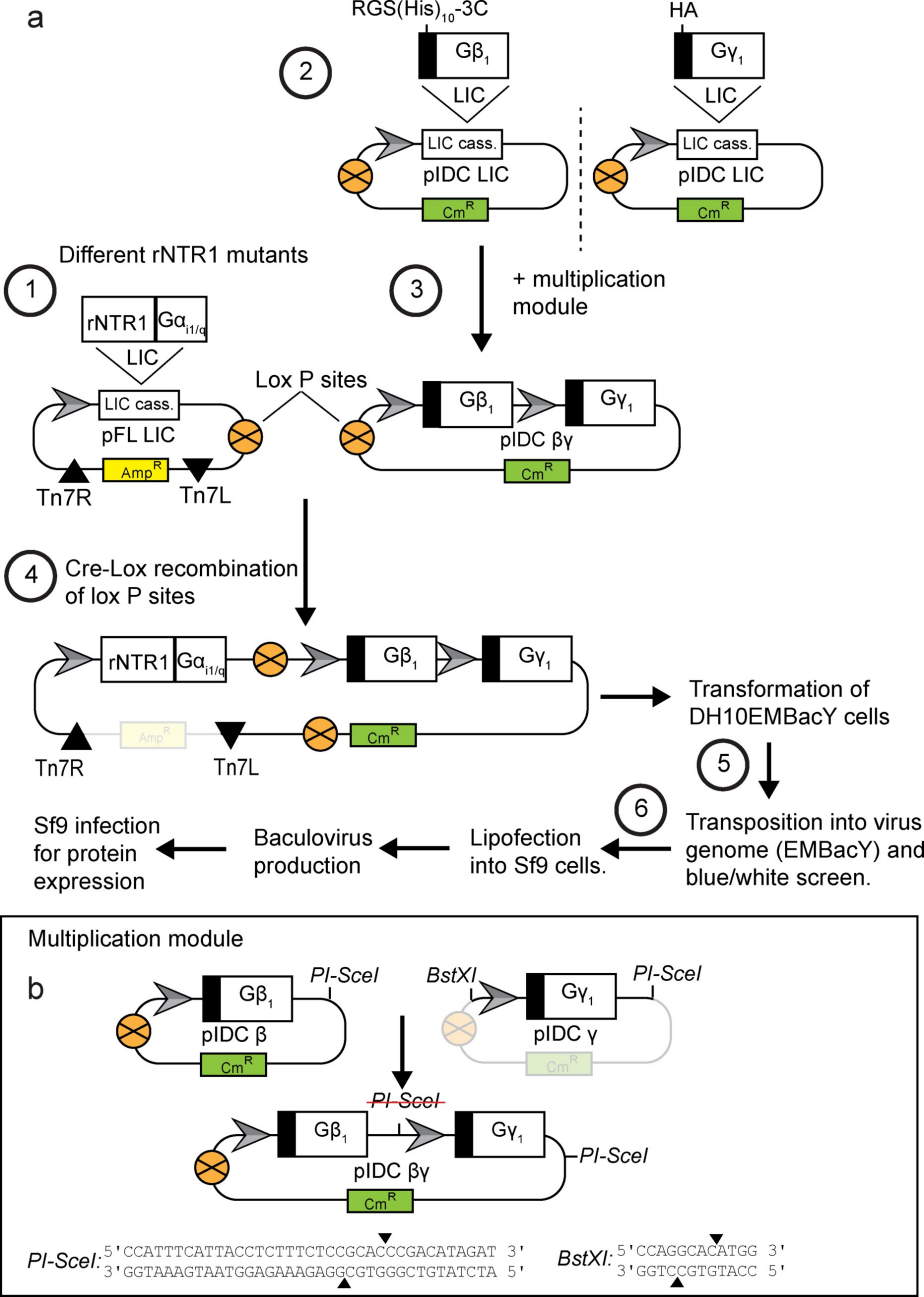
Schematic representation of the assembly of rNTR1-Gα_i1/q_, Gβ_1_ and Gγ_1_ subunits on a single baculovirus. (**a**) (1) The construct of a rat NTR1-Gα_i1/q_ fusion was cloned into the acceptor vector pFL with the help of ligation-independent cloning (LIC). (2) N-terminally RGS(His)_10_-tagged Gβ_1_ and N-terminally hemagglutinin (HA)-tagged Gγ_1_ were each cloned into an independent donor vector pIDC via LIC. (3) The Gβ_1_ and Gγ_1_ were then assembled onto a single pIDC plasmid with the help of a multiplication module (see panel b for detail), resulting in plasmid “pIDC βγ” (4) Using Lox-P sites (crossed circle, orange), an *in vitro* Cre recombination was performed. This resulted in one transfer vector on which all the three genes were present in a stoichiometry of 1:1:1, and each gene was under the control of a separate polyhedrin promotor (grey arrow). (5) DH10 EMBacY cells were transformed with the resulting transfer vector. There, the portion containing the expression modules (sequence between the two inverted black triangles) was integrated into the baculovirus genome via Tn7 transposition. (6) The virus genome was isolated and used to transfect insect cells, resulting in the formation of a first generation of baculovirus, which was used for high-level heterologous protein production. (**b**) Details of the multiplication module used to insert two (or more) genes into the pIDC vector. In the above-mentioned cloning scheme, the pIDC vector containing the Gβ_1_ subunit (“pIDC β”) was linearized using the homing endonuclease *PI-SceI*. The expression cassette (consisting of the promoter, the gene of interest and the polyadenylation site (not shown for clarity)) from the second pIDC vector containing the Gγ_1_ subunit (“pIDC γ”) was digested by *PI-SceI* and *BstXI* (recognition sequences are shown at the bottom of the panel). As the overhangs generated by digestion with *BstXI* (general recognition sequence: 5’-CCANNNNNNTGG-3’) was designed to be compatible to *PI-SceI* overhangs, the expression cassette could be inserted into the linearized “pIDC β” resulting in “pIDC βγ”. After ligation, the original *PI-SceI* site of the recipient vector was eliminated, while a new *PI-SceI* site was generated downstream of the newly inserted expression module, which, in principle, could again be used to integrate a new expression cassette.

The cloning strategy used is depicted in Fig 2 and is described in Section 4.7. We use the label rNTR1 to denote wild-type rat neurotensin receptor 1, while the mutants used are indicated using an asterisk (*) in the figure-caption.

To confirm successful integration of the plasmid into the baculoviral genome and subsequent expression of all the subunits, a western blot was performed using the transfected cells. All the subunits were found to migrate close to their theoretical molecular weights (~76 kDa, receptor-Gα fusion; ~40 kDa, RGS(His)_10_-3C-Gβ_1_; ~9 kDa, HA-Gγ_1_) (S1 Fig). A very tiny portion of possible dimer species of the receptor-Gα fusion was also observed (S1 Fig). However, the effect of any reducing agent was not investigated at this stage.

In summary, the MultiBac strategy allowed a defined homogeneous expression of all the subunits of the complex in infected *Spodoptera frugiperda* (*Sf*9) cells. More importantly, it offered a simplicity in handling by having to infect with only one virus instead of adding multiple viruses. Subsequent large-scale infections were performed with the aim of optimizing preparative scale purifications.

### 2.3 Ligand column purification

**Fig 3:**
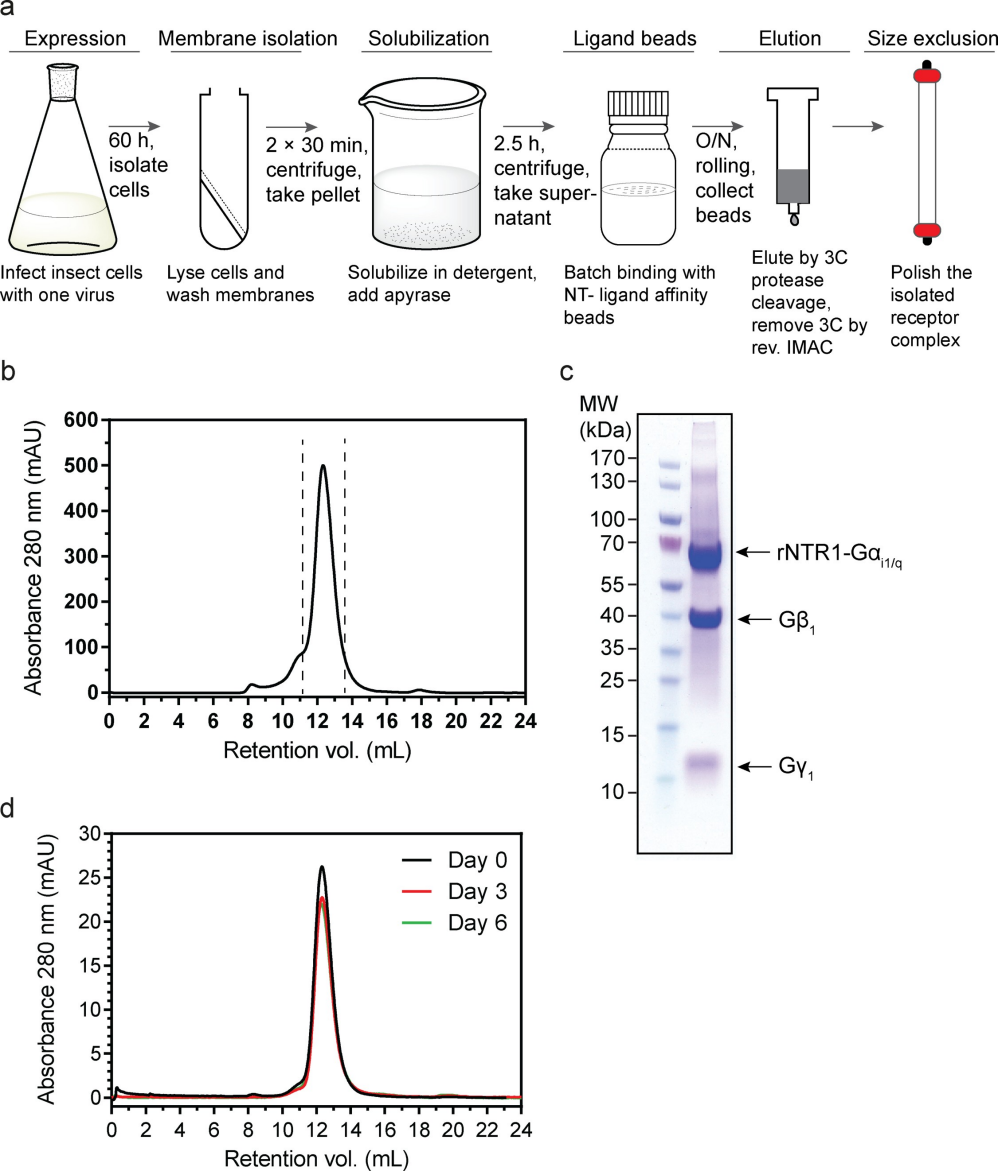
Expression and purification of rNTR1*-Gα_i1/q_β_1_γ_1_ from insect cells. (**a**) Purification scheme for the purification of rNTR1*-Gα_i1/q_β_1_γ_1_. The rNTR1-Gα_i1/q_ fusion and β_1_γ_1_ are co-expressed in insect cells using the MultiBac system, their co-expression leads to *in vivo* complex formation. After isolating the membrane, solubilization was carried out in a detergent of choice, (see text for detergents used). A ligand-affinity column was used to isolate the functional complex. The protein was then eluted from the NT ligand-affinity column using 3C protease (homemade, containing His_6_ tag) which cleaves the intact receptor-ligand complex from the column support; the 3C protease was then removed by a reverse Ni^2+^-NTA affinity column. The isolated complex was further polished using size exclusion chromatography (SEC) (**b**) Representative elution profile of purified fusion-protein complex on a Superdex 200 10/300 GL column (GE Healthcare). (**c**) SDS-PAGE Coomassie-stained gel of the pooled fractions (dashed lines in **b**) from the gel filtration column. (**d**) The stability of the purified complex was monitored by analytical SEC on the same column after incubating the protein for 6 days at 4°C. The SEC profile and SDS-PAGE image (**b** and **c**, respectively) are representative of more than fifteen independent purifications. **d** represents protein purified using an MNG:CHS detergent mixture. The asterisk (*) denotes that a mutant of rNTR1 was used: HTGH4-ΔICL3(B) (see text).

For the purification of the complex, we used the previously described neurotensin (NT)-based ligand-affinity column [26]. In this column, the Sepharose resin is coupled to the protein D (pD) from phage lambda. The protein D, in turn, is C-terminally fused to the receptor-binding NT part (residues 8–13 of NT) via a long and flexible linker that encodes an HRV 3C protease cleavable site. The ligand-affinity column enables not only a mild proteolytic elution of the ligand-receptor complex but also the isolation of only correctly folded and functional NTR1 [26]. For an *in vitro* extraction and stabilization of a GPCR–G protein complex, a high-affinity ligand is indispensable [39], and since the ligand column allows an early stabilization of the NTR1, it was initially the method of choice for the purification. The ligand-affinity-based purification scheme is shown in Fig 3A.

Purification of the receptor–G protein fusion complex was carried out from the membranes of *Sf*9 cells. Purification from membranes is beneficial as compared to starting from whole cells, as the preparation of membranes removes the cytosolic components, i.e. other proteins, proteases, and GDP/GTP that may potentially interfere with the overall stability of the complex during purification. However, unlike the previously employed protocols for the purification of unfused GPCRs from *Sf*9 membranes [40], which include a high-salt membrane wash buffer (1.0 M NaCl) to remove peripheral membrane proteins, a milder membrane wash with medium-salt buffer (0.4 M NaCl) was carried out to avoid removing Gβ and Gγ subunits. The initial attempts of purification of the fusion complex using construct TM86V-ΔICL(B)-L167R-Δ390-Gα_i1/q_ with β_1_γ_1_ were found to be low-yielding and the purified protein complex was prone to aggregation (results not shown). Therefore, to improve the yield and attain a high stability of the purified fusion complex we exchanged the receptor mutant to another NTR1 mutant, HTGH4, which has been evolved for apo-state stability [16]. An analogous deletion in the ICL3 was introduced and the C-terminal fusion position of Gα_i1/q_ was kept identical to Δ390. The final fusion construct was termed HTGH4-ΔICL3(B)-Gα_i1/q_ and it was then used to generate a fusion complex with β_1_γ_1_ by coexpression as described above. The fusion complex purified with the HTGH4 mutant showed an improved yield and had a high degree of stability.

During the initial testing of the method, a mixture of 1% n-dodecyl-β-D-maltoside (DDM) with 0.1% cholesteryl hemisuccinate (CHS) was used to solubilize the washed membranes. Subsequently, other detergents were tested and successfully used, e.g., a detergent mixture of 1% lauryl-maltose neopentyl glycol (MNG-3) and 0.1% cholesteryl hemisuccinate (CHS), or simply 1.5% n-decyl-β-D-maltoside (DM) without cholesteryl hemisuccinate (CHS) for solubilizing the membrane. Apyrase, a nonspecific nucleotide phosphatase, was added to the solubilized protein to remove any GDP and GTP that might affect the stability of the complex [39]. The mixture was then incubated with the ligand beads after removing any non-solubilized membranes. Once the protein was bound to the ligand beads, the beads were extensively washed using buffers containing the detergent of choice (see S1 Table). Incubation with catalytic amounts of 3C protease led to the elution of ligand-bound receptor-Gα fusion as a complex with Gβγ (see S2 Fig for SDS-PAGE analysis of different steps of the purification). The co-eluted 3C protease was removed by a reverse Ni^2+^-NTA by virtue of its His_6_ tag. The pure receptor–G protein complex was evaluated by quantitative size exclusion chromatography (Fig 3B). Typically, a highly-monodisperse protein preparation was obtained. As observed by the SDS-PAGE analysis of the pooled fractions, the purity of the complex was >95% (Fig 3C). In order to confirm the long-term monodispersity of the purified complex, we performed analytical SEC of the complex following incubation at 4°C for up to 6 days. The monodisperse nature of the purified complex was preserved upon incubation (Fig 3D).

The detergent exchange could be successfully performed on the ligand-affinity column (see S3 Fig for a compilation of further SEC profiles using alternative detergents). The typical yield of the purified HTGH4-ΔICL3(B)-Gα_i1/q_β_1_γ_1_ complex in DDM:CHS was 1.0–1.5 mg per liter of insect cell culture. The yield was similar in other tested detergents. For exchanging into short-chain detergents, we observed that DM is a better starting detergent for solubilizing the complex compared to DDM:CHS.

Our previously described *in vitro* assembly of NTR1 –G protein complex made use of separately purified components, but the purification procedures for individual components are laborious and subsequent assembly of the complex requires preparing an equimolar mixture of individual components, followed by a long dialysis, thus making the entire process very work-intensive [18]. The MultiBac assembly of the NTR1 –G protein fusion complex and the ligand-affinity chromatography greatly reduced the effort and the time taken for expression and purification of the NTR1 –G protein fusion complex. As noted before, during infection of insect cells only a single virus (instead of multiple viruses) was needed for obtaining a uniform expression of all components of the fusion complex. Additionally, the ligand-affinity column simplified the purification procedure, as in rather few steps the fusion complex can be efficiently isolated to a very high purity. The ligand-affinity purification method offered a highly reproducible and fast technique with which milligram quantities of the purified fusion complex could be easily generated within one working day.

### 2.4 Complex activity and stability

**Fig 4:**
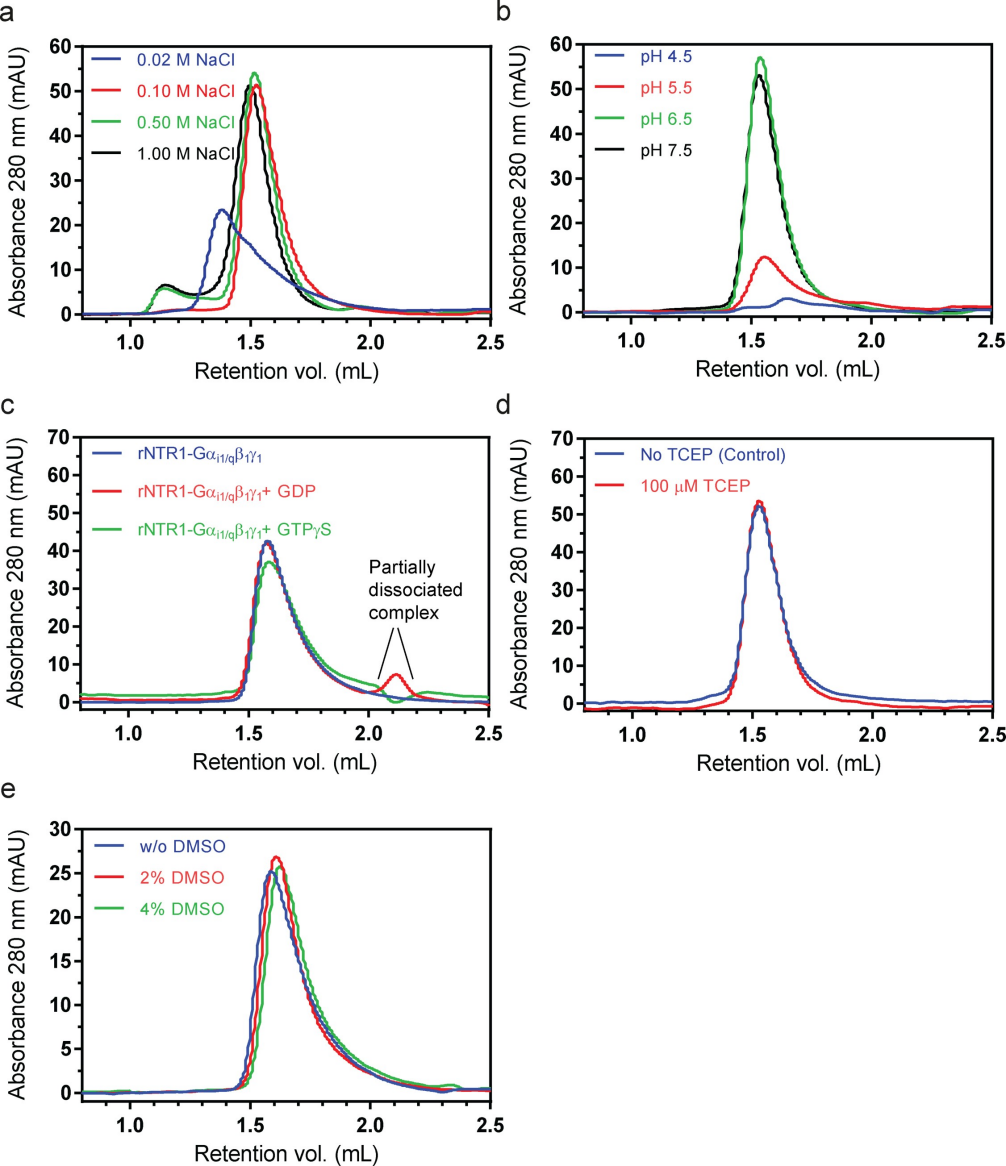
Effect of ionic strength, pH, nucleotide analogs, DMSO concentration, and TCEP on the stability of the rNTR1*-Gα_1i/q_β_1_γ_1_ complex. (**a**) Analytical gel filtration showing the NaCl tolerance of the complex. The protein mixture was incubated for three days at 4°C. The complex was stable at 100 mM and eluted as a prominent monodisperse peak, but at 500 mM and 1 M NaCl, an aggregation peak was seen. Lower salt concentration (20 mM NaCl) affected the monodispersity of the complex. (**b**) The effect of various pH values was probed using analytical gel filtration, after incubating the protein for three days at 4°C in a buffer adjusted to the given pH value. Acidic pH at 5.5 or below was deleterious to the stability of the complex. (**c**) GDP and GTPγS analogs (100 μM, final concentration) were mixed with the purified complex and the mixture was incubated overnight at 4°C; as it can be seen, the nucleotide analogs caused a partial dissociation of the complex. The excess nucleotides in the buffer migrate at about 2.7 mL on the gel filtration column (not shown). (**d**) Purified protein was incubated without or with 100 μM TCEP for up to seven days at 4°C, however, there was no change of the monodisperse peak under either condition. (**e**) The effect of increasing concentrations of DMSO (2% and 4% (v/v), final concentration) was also tested; the mixture of DMSO and purified protein was incubated for a period of three days at 4°C. The stability of the complex was not affected by up to 4% (v/v) DMSO. All the analytical gel filtrations were performed on a Superdex 200 Increase 5/150 GL column (GE healthcare). The SEC profiles are representatives of experiment(s) performed once (**a, b**) or twice (**c, d** and **e**). * rNTR1 mutant used: HTGH4-ΔICL3(B).

Structural characterizations of proteins not only require a sufficient amount of the purified protein but also require them to be in a well-folded and functional state. Therefore, we checked the influence of various components in the buffer on the stability of the purified protein using analytical SEC (Fig 4). Fig 4A shows the effect of ionic strength, using NaCl at various concentrations, on the purified complex. We observed that the monodispersity of the protein was lost at low concentrations (20 mM) of NaCl. Additionally, we found an aggregation peak in the elution profile when the protein was incubated at high concentrations of NaCl (500 mM of NaCl and 1 M of NaCl). However, at an optimal concentration (100 mM) of NaCl the protein retained a high degree of monodispersity.

The effect of the buffer pH values was also probed (Fig 4B) and the result indicated that acidic pH (5.5 or lower) caused a significant loss of the stability of the protein. We also tested the purified HTGH4-ΔICL3(B)-Gα_i1/q_β_1_γ_1_ complex for dissociation upon binding to the non-hydrolyzable GTP analogue GTPγS (Fig 4C). As can be seen, nucleotides GDP and GTPγS (100 μM) caused only a partial dissociation of the complex. This result was surprising, since purified GPCR–G protein complexes have been shown to dissociate completely at 10 μM and at 100 μM GTPγS concentration in the case of Rhodopsin-Gt complex [41] and the β_2_AR-Gs complex [39], respectively. The unusual nucleotide characteristics of G protein in complex with the evolved NTR1 variants has been observed previously [18]. In co-immunoprecipitation experiments, the interaction of the evolved NTR1 mutants (TM86V-L167R) with the G protein was only partially disrupted even with a high concentration of GTPγS (750 μM used in Hillenbrand et al. [18]). In contrast, wild-type NTR1 exhibited significant dissociation already at 0.1 μM GTPγS, while the purified complex of the evolved NTR1 mutants (TM86V ΔIC3A) with Gα_i1_β_1_γ_1_, following an overnight incubation with 100 μM GTPγS, did not exhibit complete dissociation [18]. We have used here the HTGH4 mutant of NTR1, which has been evolved for apo-state stability, to generate the fusion complex. The evolved mutants can form a stable complex even in the presence of GTPγS, presumably due to conformational trapping within the G protein in the complex. Additionally, as the Gα is fused to the receptor, only Gβγ can possibly dissociate. Thus, the fact that incubation with GTPγS does not lead to a complete dissociation of complex may in turn prove valuable for future studies. Additionally, in the absence of tris(2-carboxyethyl)phosphine hydrochloride (TCEP) no cysteine-mediated cross-linking or dimerization of the purified complex was observed for up to 7 days (Fig 4D).

Dimethyl-sulfoxide (DMSO) is often used during co-crystallization of proteins in complex with ligand(s) of lower aqueous solubility [42]. Since many GPCR ligands, allosteric modulators, and G protein inhibitors exhibit a lower aqueous solubility [43, 44], we tested the stability of the purified complex at various concentrations of DMSO for up to three days (Fig 4E). As it can be seen, the monodisperse nature of the purified protein complex was preserved for up to 4% (v/v) DMSO. The experiment can be a guiding tool for designing experiments for co-crystallization or cryo-EM investigations with a ligand having low aqueous solubility. In summary, the approaches and the general findings described here may be used in future studies that aim to perform general biophysical and stability assessment of other purified GPCR–G protein complexes.

### 2.5 GFP-DARPin-based generic purification method

While the ligand-affinity method provides an excellent approach to purify functional NTR1 or NTR1 –G protein complex, the method is not generic. Additionally, the method requires NTR1 mutants that can be solubilized from the membrane and remain functional in the apo-state. Although the evolved NTR1 mutants (e.g. HTGH4) can tolerate a long solubilization in an apo-form, the same is not true for the majority of GPCRs, many of which require ligand to be present from the early stage of solubilization and purification. Thus, purification of analogous GPCRs–G protein fusion complexes would require other efficient purification methods. Even though an anti-FLAG affinity column has been used for the purification of a receptor-G protein complex for cryo-EM-based structure determination [33], the method is very tedious and expensive.

We thus aimed to generalize the purification strategy for other GPCR–G protein fusion complexes. For this purpose, we utilized a novel high-affinity resin based on a Designed Ankyrin Repeat Protein (DARPin) clamp, wrapping around Green Fluorescent Protein (GFP) [27]. The resin is covalently coupled to a DARPin that binds to the GFP (or variants thereof, such as YFP and eYFP) with a very high affinity and a high specificity, thus allowing pull down of proteins bound to GFP (or variants thereof) in a single step. This resin is very inexpensive to prepare. We fused a 3C-protease-cleavable enhanced Yellow Fluorescent Protein (eYFP) to the N-terminus of the Gγ_1_ subunit. To compare the efficiency of GFP-DARPin affinity purification with the previously described NT ligand-affinity-based pull-down method, the NTR1 mutant, NTSR1-EL [45] was chosen as the test GPCR in the fusion complex. NTSR1-EL is characterized to be a constitutively active mutant [45] having only four mutations. This mutant can be expressed in insect cells, but because of its lower stability than HTGH4 it cannot be purified in the apo state. Therefore, it requires an early stabilization of the receptor by adding the NT_8-13_ ligand before its extraction from the membrane, and can then be purified with the established histidine tag-based purification method.

**Fig 5:**
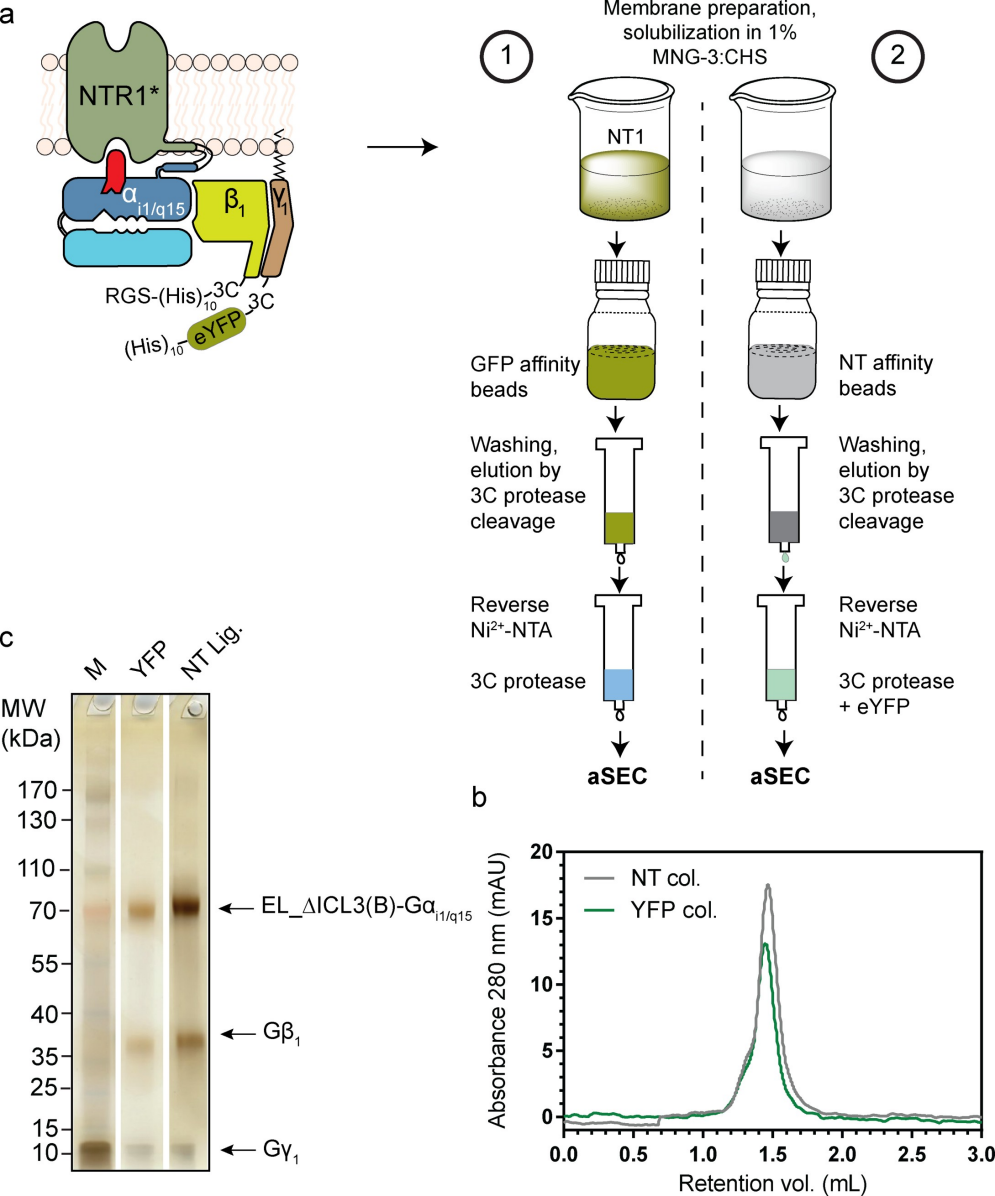
Comparison of GFP (or variant) affinity and ligand affinity purification. (**a**) Schematic diagram of the construct used for comparing the GFP trap and the ligand pull-down methods. The NTSR1-EL mutant with deleted ICL3 (named EL _ΔICL3(B)) was fused to Gα_i1/q15_, a 3C-cleavable RGS-decahistidine tag was present at the N-terminus of Gβ_1_, while a deca-histidine tag followed by an enhanced YFP (eYFP) followed by a 3C protease cleavage site was present on the N-terminus of Gγ_1_. Prepared membranes were separated into two fractions. Numbers denote two schemes of purification. 1 –GFP-affinity scheme: membranes were solubilized in the presence of NT1 (a variant of NT_8-13_, see Section 4.9); batch binding with GFP-affinity beads; after washing elution was done using cleavage by 3C protease, the eYFP moiety was retained on the column while a mixture of protein and 3C protease was eluted; reverse Ni^2+^-NTA removed the co-eluted 3C protease; the flow-through was used for analytical SEC (aSEC). 2 –ligand-affinity scheme: membranes were solubilized in the absence of NT1; batch binding with NT-ligand affinity beads, after washing elution was done using cleavage by 3C protease; the mixture of protein, eYFP and 3C protease was eluted; reverse Ni^2+^-NTA removed the eYFP and the 3C protease; the flow-through was used for aSEC. (**b**) Comparison of aSEC runs of protein from the two columns. (c) The highest protein-containing fraction from aSEC was analyzed using SDS-PAGE followed by silver staining. Lanes: M- molecular weight marker, YFP- protein fraction for the GFP column eluate. NT lig.—Protein from the ligand-affinity column. *rNTR1 mutant used NTSR1-EL-ΔICL3(B).

We created an NTSR1-EL-Gα fusion construct and used the previously described MultiBac system to generate an *in vivo* complex with the recombinant Gβ and Gγ subunits. A schematic diagram of the construct used is shown in Fig 5A, and described in Section 4.11. We compared the two purification methods (Fig 5). In short, unlike the membrane solubilization for the NT ligand-affinity method, the solubilization for the GFP-affinity-based method was carried out in the presence of NT peptide. After removing the membrane fraction that could not be solubilized, the soluble portions were incubated with the respective bead types. The remaining procedure of the two methods is similar and is explained in Fig 5 and Section 4.12. Following incubation with 3C protease, a mixture of protein complex and 3C protease was eluted, while the eYFP moiety was retained on the GFP-DARPin column. The eluate from the ligand column contained a mixture of protein complex, eYFP and 3C protease. Reverse Ni^2+^-NTA columns were then used to remove 3C protease (co-eluted from the GFP-DARPin column) or a mixture of cleaved eYFP moiety and 3C protease (co-eluted from the ligand-affinity column) by virtue of their respective histidine-tags.

The purified protein was analyzed using analytical SEC (Fig 5B). As can be observed, similar to the protein purified using the ligand-affinity-based method, the protein purified using the GFP-affinity-based method migrated mainly as a sharp peak with only a minor shoulder, indicating the monodisperse nature of the complex. The calculated molecular weight of the peak fraction on aSEC was 232 kDa (GFP-DARPin column) and 219 kDa (ligand-affinity column), which is within experimental error of each other. The theoretical molecular weight of the complex was 125.2 kDa after the truncation of the eYFP and the histidine-tags; however, the protein complex is surrounded by a detergent micelle and distinctly non-globular, and thus the estimated molecular weight is consistent with the molecular composition. The high purity of the proteins from the two columns can be judged by the silver-stained SDS-PAGE gel (Fig 5C), and was found comparable.

Thus, the performance of the GFP-DARPin column was found to be similar as that of the ligand-affinity column. While the ligand affinity columns secure the purification of active receptor, the GFP-DARPin affinity purification strategy can be carried out in the presence of any ligand. The GFP-DARPin column may have some advantages as it provides a generic, inexpensive, and simple strategy for the purification of similar fusion complexes. Additionally, the inclusion of ligand during the membrane preparation and solubilization step can provide a very early stabilization of the receptor–G protein complex and therefore should assist in a better extraction of less stable complexes from the membrane. Moreover, as the protein remains bound to the affinity bead, a detergent exchange step can be very efficient. The GFP-DARPin column material is very easy and inexpensive to be generated and offers a high capacity and reusability, and the bound GFP (or variant) can be eluted and the clamp is regenerated [27].

## 3. Conclusions

Structural and functional studies of GPCRs and their complexes with transducer proteins are vital in understanding the mechanism of action of this important class of proteins. While at the time of writing there had been only one crystal structure of a GPCR with a heterotrimeric G protein, several GPCRs and their complexes with G proteins have been structurally characterized using cryo-EM. However, the nature of interactions between many pharmaceutically important GPCRs with other G proteins remain elusive. Additionally, the nucleotide-free complexes utilized in previous structural investigations are expected to be distinct from the GDP- and GTP-bound complexes. In-depth analysis of GPCR–G protein complexes in the presence of various allosteric modulators, biased-ligands and nucleotides will provide important insight into the G protein coupling, specificity and aspects of biased signaling. Thus, a rapid assembly and high quality of purified complexes is of paramount importance in such efforts.

In this report, we intended to generate milligram quantities of receptor–G protein complex using a receptor-Gα fusion strategy. Although artificial, such fusions represent attractive alternative targets for structural and functional studies given their historically successful use in studying pharmacological aspects of receptor–G protein interactions. Analogous fusion proteins have been tested for their purification, although without the βγ heterodimer [23]. Our study proposes the use of receptor-Gα fusion as an alternative approach for the production of high quality receptor–G protein fusion complexes. This study demonstrates that by using the baculovirus-based MultiBac system, the designed NTR1-Gα fusion can be used to form an *in vivo* functional complex with Gβγ, resulting in the generation of an entire NTR1 –G protein complex. The complex can be successfully purified in a functional form using a ligand-affinity method. Moreover, we probed the stability of the purified complex under various conditions. The results may serve as guiding tools for future experiments for similar characterization of other GPCR–G protein complexes and subsequent attempts for their structural studies.

Furthermore, we could also show the use of a GFP-affinity-based generic purification strategy. The method was successfully applied to a less stable wild-type-like NTR1 –G protein fusion complex. The efficiency of the GFP-affinity-based purification was found to be comparable to the ligand-affinity-based method. The GFP-affinity-based purification method is an inexpensive generic method and may easily be applied to other GPCR-Gα fusion complexes, and many other complexes, for their efficient purification.

Taken together, the ease of assembly of fusion complexes using the MultiBac system and the generic purification procedure described in this study will assist the undertakings aiming at understanding the structural and functional basis of the modular interactions of pharmacologically important yet unexplored GPCRs and their complexes.

## 4. Materials and methods

### 4.1 Materials

All the material/chemicals used were of the highest quality and were purchased from Sigma or AppliChem. Enzymes for molecular biology were from New England Biolabs or Thermo Fisher Scientific. Anatrace was the primary supplier for all the detergents used, except for cholesteryl hemisuccinate Tris salt (CHS), which was purchased from Sigma. DNaseI was purchased from Roche. Empty disposable PD-10 columns, Superdex 200 10/300 GL, and Vivaspin protein concentrators were purchased from GE Healthcare. The tritiated agonist [^3^H] neurotensin and [^35^S]GTPγS (1,250 Ci/mmol) was purchased from Perkin-Elmer. The unlabeled truncated neurotensin ligand (NT_8-13_, RRPYIL) and its variant NT1 (GPGGRRPYIL) were purchased from Anaspec.

### 4.2 Constructs design for expression of NTR1-Gα fusion proteins in *Escherichia coli*

The modified pRG vector [29] was used to generate all NTR1-Gα fusion constructs for all bacterial expressions. The open reading frame of the longest construct (Δ421-TEV-(N_5_)-(G_3_S)_2_-Gα_i1/q_) encoded the N-terminal maltose binding protein of *E*. *coli* with its own signal sequence (MBP, Lys1 to Thr336), followed by glycine, serine, a hexa-histidine tag, a tobacco etch virus (TEV) protease cleavage site, a *Bam*HI cloning site, N-terminally truncated TM86V_L167R[17] (Thr43 to Arg420), a TEV protease cleavage site, a (N_5_)-(G_3_S)_2_ linker, an *Eco*RI cloning site and the chimeric Gα_i1/q_ (Gly2 to Val*354), where the asterisk denotes that this is a non-native residue. Gα_i1/q_ refers to the chimeric Gα_i1_, where the five C-terminal amino acids of human Gα_i1_ (Uniprot–P63096, amino acids Asp^H5.22^ to Phe^H5.26^) were exchanged to those of human Gα_q_ (Glu^H5.22^ to Val^H5.26^). Here, the superscripts refer to the Common Gα Numbering (CGN) scheme [46]. Thus, Val*354 in the chimeric Gα_i1/q_ refers to the exchanged C-terminus Val359 of the human Gα_q_ (Uniprot–P50148). Before generating any shorter construct, a modified variant of the original plasmid containing the longest construct (Δ421-TEV-(N_5_)-(G_3_S)_2_-Gα_i1/q_) was designed. This was done to simplify cloning and making purification of shorter constructs efficient, which was required in the initial phase of the project. This modified plasmid had the following changes compared to the longest construct, (1) the TEV protease site N-terminal to the receptor (preceding the *Bam*HI site) was exchanged to HRV 3C protease site; (2) a *Bam*HI and *Eco*RI flanked *sacB* [47] gene was introduced in place of NTR1 as a negative selection marker; (3) the TEV protease site and the (N_5_)-(G_3_S)_2_ linker present between C- terminal of receptor and *Eco*RI site (preceding the Gα_i1/q_) was removed. Thus, using the modified plasmid the *sacB* gene could readily be exchanged with truncated receptor genes using conventional *Bam*HI and *Eco*RI cloning. The initial assessment of the optimal fusion positions was done with an evolved NTR1 mutant, TM86V_L167R [17]. The *sacB* gene was replaced with genes encoding for C-terminally truncated variants of TM86V_L167R, thereby generating in-frame fusion constructs with Gα_i1/q_. The receptor was truncated initially, in a step of five residues, and then a narrow screen of one residue at a time. The first missing amino acid is denoted after “Δ” in the truncation constructs. The *E*. *coli* strain XL1-blue (Agilent) was used for all the cloning purposes and, after the confirmation of the correct gene sequence, the expression plasmid was used for transformation of the *E*. *coli* expression strain BL21 (DE3).

### 4.3 *E*. *coli* expression and membrane preparation

*E*. *coli* BL21 (DE3) cells, transformed with the expression plasmid, were grown in 2YT medium supplemented with ampicillin (100 μg/ml) and 0.2% (w/v) glucose. Protein expression was induced by adding 1 mM of isopropyl-β-D-1-thiogalactopyranoside (IPTG) upon reaching an optical density of 0.6 at 600 nm. After incubation for 22–24 h at 20°C, cells were harvested. Spheroplasts were prepared as in Witholt et al. [48] and lysed by osmotic shock in ice-cooled water. The unlysed cells were disrupted using gentle sonication at 4°C in buffer A (50 mM Tris-HCl pH 8.0, 1 mM EDTA). Complete protease inhibitor tablets (Roche) were used throughout the process of cell-lysis and membrane preparation. Membranes were isolated by ultra-centrifugation (66,000× g for 1 h at 4°C), and re-suspended in buffer A with 20% (w/v) sucrose. The total membrane protein concentration was measured using Quant-iT assay kit (Invitrogen). Membranes were flash-frozen in liquid nitrogen and stored at −80°C.

### 4.4 Radio-ligand binding assay on rat NTR1- Gα_i1/q_ fusions expressed in *E*. *coli*

Appropriate amounts of membrane preparations were diluted in buffer (50 mM Tris-HCl pH 7.4, 1 mM EDTA, 0.1% (w/v) bovine serum albumin (BSA), 40 μg/mL bacitracin), 10 nM [^3^H]-NT (final concentration) (cat. no. NET605025UC, PerkinElmer) was added and the suspension was incubated for 2 h at 4°C. The reaction mixture was transferred to a 96-well MultiScreen glass fiber filter plate (Millipore), which was pretreated with a 0.5% polyethyleneimine (PEI) solution. The filters were washed five times with 200 μl of ice-cold wash buffer (50 mM Tris-HCl pH 7.4), and transferred to a 96-well scintillation plate, dissolved overnight in 200 μl Optiphase Supermix scintillation cocktail (PerkinElmer) and the filter-bound radioactivity was counted using a 1450 MicroBeta plus counter (PerkinElmer). Non-specific binding was measured in the presence of 10 μM NT_8-13_ (Anaspec). Data from each experiment were normalized to the amount of crude membrane protein present in the membrane preparations.

### 4.5 [^35^S]GTPγS binding assay on rat NTR1- Gα_i1/q_ fusions expressed in *E*. *coli*

Membranes containing a defined number of receptor fusions (determined by radioligand binding assays) were diluted (final concentration 1.5 nM) in the assay buffer (50 mM Tris-HCl pH 7.4, 5 mM MgCl_2_, 50 mM NaCl, 1 mM EDTA, 0.1% (w/v) BSA, 0.1 mM DTT, 1 μM 1.10-phenanthroline and 3 nM GDP). To this mixture, either no ligand or 200 μM of NT_8-13_ were added. After pre-incubation (15 min, 28°C), [^35^S]GTPγS (cat. No. NEG030H250UC, 1,250 Ci/mmol, PerkinElmer) was added to a final concentration of 0.1 nM and the mixture was further incubated while shaking for 45 min at 28°C. The reaction was stopped by filtration through pre-soaked (in UHP) 96-well MultiScreen glass fiber filter plates (Millipore) and by washing five times with 200 μl of ice-cold wash buffer (50 mM Tris-HCl pH 7.4, 5 mM MgCl_2_, 50 mM NaCl, 1 mM EDTA). Filters were treated as in the radio-ligand binding assay and the filter-bound radioactivity was determined by liquid scintillation counting. Data from each experiment were normalized to the amount of receptor present in the membrane preparations; graphs were prepared using Prism 6.0 (GraphPad).

### 4.6 Generation of cleavable NT ligand-affinity column

The method for generating a cleavable NT ligand-affinity column was as described by Egloff et al. [26].

### 4.7 Constructs for MultiBac assembly and Baculovirus generation

The component vectors of MultiBac system [38, 49], the baculovirus donor vector (pIDC) and acceptor vector (pFL) were a kind gift from Imre Berger [European Molecular Biology Laboratory (EMBL), Grenoble, France]. In earlier work [18], the pIDC and pFL had been modified to contain a *Bss*HII-cleavable *sacB* negative selection marker [47], along with a ligation-independent cloning (LIC) cassette, which places the gene of interest under the control of the polyhedrin promoter.

For working out the purification method, we utilized another rat NTR1 mutant, HTGH4 [16] (Gly50 to Pro389) and, analogous to the TM86V-ΔICL3(B) construct, we created an ICL3 deletion construct (ΔICL3(B): residues Glu273 to Thr290 deleted). The construct was named HTGH4-ΔICL3(B) and we used it to generate a receptor-Gα fusion as before. The chimeric Gα_i1/q_ was identical as described before and it was fused to the analogous C-terminal residue (Pro389 as in the construct Δ390-Gα_i1/q_) that was found to be optimal for a functional receptor coupling using the [^35^S]GTPγS assay. The final fusion construct was named HTGH4-ΔICL3(B)-Gα_i1/q_.

A pIDC vector containing the N-terminally 3C-cleavable MRGSHis_10_-tagged human Gβ_1_ (Uniprot–P62873) was a kind gift from Matthias Hillenbrand [University of Zürich, Switzerland]. Additionally, the N-terminally hemagglutinin (HA)-tagged human Gγ_1_ (Uniprot–P63211) was amplified using primers containing overhangs for LIC sites of the pIDC vector (forward primer: 5′-CGAAACAAAGCGCGTTACCATGTACCCATACGATG-3′; reverse primer: 5′- ACGAAGACGCGCGTTTATGAAATCACACAGCC-3′). The GPCR-Gα fusion genes were amplified with primers containing overhangs for LIC sites for the pFL vector (forward primer: 5’-TACATTTCTTACATCTAT**GCG**GGTCCGGGATCCGG-3′; and reverse primer: 5’-TTACCAATACTTAAGTTAGACCAGATTGTACTCC-3′). This procedure places the GPCR-Gα fusion gene under the control of the polyhedrin promoter, preceded by an N-terminal melittin signal sequence. The bold **GCG** encodes the last Ala of the melittin signal sequence.

The *Bss*HII-linearized vector and PCR products were treated with T4 DNA polymerase in the presence of dTTP or dATP, respectively. Treated vector and PCR products were annealed, and *E*. *coli* strains BW23474 [50] (for pIDC vectors) or XL1-blue (for pFL and pFL/pIDC fusions) were transformed with it and then cultured in the presence of 7% (w/v) sucrose (*sacB*-dependent negative selection). Subunits Gβ_1_ and Gγ_1_ were subsequently assembled on a single pIDC vector with the help of the multiplication module (Fig 2B). The GPCR-Gα fusion gene was placed on a pFL vector. Both vectors, pIDC and pFL, containing the different subunits, were then fused by Cre recombinase, making use of the *loxP* sites on the vectors. The 1:1 stoichiometry of both vectors in the final transfer vector was checked by *Age*I digestion. A representative vector map of the final Cre-Lox recombined vector is shown in S4 Fig. The *E*. *coli* DH10EMBacY cells [38] were transformed with the final transfer vector containing genes for the GPCR-Gα fusion, Gβ and Gγ. The baculovirus genome was isolated and used for transfection of *Spodoptera frugiperda* (*Sf*9) cells as described [51]. The resulting virus was passaged twice before using it for expression. The amplified virus was used directly for small-scale expression (~50 mL). For large-scale expression (>3 L) Baculovirus Infected Insect Cells (BIIC) [52] were prepared. For preparing a BIIC stock, 50 mL of *Sf*9 cells at a density of 1× 10^6^ cells per mL were infected with an MOI of five. After 24 h, the cells were collected by centrifugation (200× g for 10 min). The cells were resuspended in 5 mL of freshly prepared freezing medium (SF900 II medium with penicillin-streptomycin +10% (v/v) dimethyl-sulfoxide (DMSO)), filled at 5× 1 mL into cryo vials and frozen with an optimal freezing rate of −1°C/min. The vials were stored in a −80°C freezer for a day and shifted to a −150°C freezer for long-term storage. One mL of frozen BIIC is theoretically sufficient for 3.3 L of expression culture. The actual volume of BIIC stock used for infection was empirically determined (2–5× the theoretical value was found optimal).

### 4.8 Insect cell expression

*Sf*9 cells in SF900 II medium (Thermo Fisher Scientific Inc.) were grown in suspension at 27°C with shaking (90 rpm, in an orbital shaker). For small-scale expression, cells were infected with a MOI of five in shake flasks at a density of 3 × 10^6^ cells per mL. After 72 h post infection, cells were harvested by centrifugation and stored at −80°C. For large-scale expression, Baculovirus Infected Insect Cells (BIIC) [52] were used for infection. Cells were harvested at 96 h post infection and stored at −80°C.

### 4.9 Purification of rat NTR1-Gα_i1/q_ β_1_γ_1_ by cleavable NT ligand column

*Sf*9 cell pellets were lysed by homogenization in lysis buffer (10 mM HEPES pH 7.5, 1 mM EDTA) supplemented with 100 μM 4-(2-aminoethyl)benzenesulfonyl fluoride hydrochloride (AEBSF), 1 μM leupeptin, 50 μg/mL DNaseI. Membranes were washed by homogenization in membrane wash buffer (10 mM HEPES pH 7.5, 1 mM EDTA, 400 mM NaCl) supplemented with 100 μM AEBSF and 1 μM leupeptin, and collected by centrifugation at 95,800× g for 30 min. For membrane solubilization and purification, various detergents at concentrations depending on their critical micelle concentration were used as summarized in S1 Table. In the text below, we have used “detergent of choice” to refer to the used detergent.

The membranes were solubilized in 50 mM HEPES pH 7.5, 20% glycerol, 200 mM NaCl, 2 mM MgCl_2_, detergent of choice (concentration as mentioned in S1 Table), supplemented with 100 μM AEBSF, 1 μM leupeptin, and apyrase (25 mU/mL) for 2.5 h at 4°C. The insoluble materials were removed by ultra-centrifugation at 95,800× g for 30 min. The supernatant was supplemented with additional apyrase (final concentration 50 mU/mL), mixed with 2.5 mL of equilibrated ligand-affinity beads (2.5 mL of beads for 1 L of starting *Sf*9 culture), and kept rolling for overnight at 4°C. The mixture was centrifuged at 200× g for 5 min and the beads were poured in an empty PD-10 column. The beads were then washed with 25 column volumes (CV) of wash buffer-1 (25 mM HEPES pH 7.5, 600 mM NaCl, 10% glycerol, detergent of choice, 100 nM NT1) and washed again with 15 CV of wash buffer-2 (25 mM HEPES pH 7.5, 150 mM NaCl, 10% glycerol, detergent of choice, 100 nM NT1). The beads were then re-suspended in wash buffer-2 (a single CV) and purified 3C protease (~500 μg in a volume of ~300 μl, homemade) was added. The column was closed at both ends, and the mixture was incubated for 2 h while rolling at 4°C. After incubation, the protein was collected by applying wash buffer-2 (typical volume of elution was 3–4 times CV). The co-eluted 3C protease was then separated from the purified protein using a reverse Ni^2+^-IMAC purification. The sample was concentrated to 0.25 mL and loaded onto a Superdex 200 10/300 GL (GE healthcare), pre-equilibrated with size-exclusion buffer (10 mM HEPES pH 7.5, 150 mM NaCl, detergent of choice, 100 nM NT1). Here, NT1 corresponds to the C-terminal part of NT, which is cleaved off the ligand-affinity column by HRV 3C protease, and stays in the ligand binding site of the receptor. NT1 thus consists of the remaining part of the HRV 3C protease site (GP), two linker residues (GG) and the NTR1 binding-epitope of NT, which is NT_8-13_ (RRPYIL). The final yield of the purified complex was 1.0–1.5 mg L^-1^ insect cell culture. The purity of the protein sample was analyzed using SDS-PAGE.

The stability of the purified protein complex was determined by keeping the sample at 4°C and running it onto a Superdex S200 GL10/300 gel filtration column on day 3 and day 6.

### 4.10 Analytical size-exclusion chromatography

For measuring the effect of various components in the buffer on the purified protein complex, the following procedure was used. Solutions of purified protein complex at concentrations ranging from 2–4 μM (concentration was identical for testing a given set of conditions) in a volume of 100 μl of SEC buffer (10 mM HEPES pH 7.5, 150 mM NaCl, 0.28% NG, 100 nM NT1), adjusted to the specified conditions or supplemented with the specified components as described in Fig 4, were prepared. For testing the effect of GDP and GTPγS, the solutions were also supplemented with 0.1 mM MgCl_2_. The solutions were incubated at 4°C for a time-period ranging from overnight to seven days, depending on the conditions and as described in Fig 4. Prior to loading on the column, the mixtures were centrifuged at 10,000× g for 10 min at 4°C. The analytical SEC runs were performed on a Superdex 200 Increase 5/150 GL column (GE Healthcare) pre-equilibrated in the same buffer. The elution profile was monitored by absorption at 280 nm.

### 4.11 Construct for the GFP-DARPin-based purification

The N-terminally FLAG-tagged mutant of NTR1, named NTSR1-EL [45] (Thr43 to Lys396, containing mutations Ala186Leu, Gly215Ala, Phe358Ala and Val360Ala) was modified by deleting the ICL3 (ΔICL3(B): residues Glu273 to Thr290 deleted). The ICL3-deleted construct, referred to as EL_ΔICL3(B), was used to create a fusion with Gα_i1/q15._ The final fusion construct was termed EL_ΔICL3(B)-Gα_i1/q15_. Here, Gα_i1/q15_ refers to the chimeric Gα_i1_, where the fifteen C-terminal amino acids of human Gα_i1_ (Thr340^H5.12^ to Phe354^H5.26^) were exchanged to those of human Gα_q_ (Lys345 ^H5.12^ to Val359 ^H5.26^). In a separate study, we had found that the EL_ΔICL3(B) construct formed a stable complex with the chimeric Gα_i1/q15_. A 3C-cleavable RGS (His)_10_ tag was N-terminally placed on Gβ_1_. A (His)_10_ tag, followed by an enhanced Yellow Fluorescent Protein (eYFP), followed by a 3C protease cleavage site was placed N-terminally on Gγ_1_. The genes for the EL_ΔICL3(B)-Gα_i1/q15_ fusion and the recombinant Gβ_1_ and Gγ_1_ were assembled on a single acceptor vector using the earlier described MultiBac system. The schematic diagram of the construct is shown in Fig 5A.

### 4.12 Purification of the rat NTR1-Gα_i1/q_ β_1_γ_1_ by the GFP-DARPin column

The GFP-DARPin affinity column material was a kind gift from Santiago Vacca [University of Zürich, Switzerland] and was prepared as per the established protocol [27]. The membrane pellet corresponding to 0.8 L of insect cell culture was resuspended in 1.6× solubilization buffer (80 mM Tris-HCl pH 7.4, 48% (v/v) glycerol, 32 mM EDTA). The resuspended membranes were split in two fractions of equal volumes, one corresponding to the ligand affinity column and the other for GFP affinity column. For the ligand affinity column, detergents and salts were added to their final concentrations, 1% (w/v) MNG-3/0.1% (w/v) CHS and 200 mM NaCl, respectively. The mixture was homogenized using a hand-held glass homogenizer. For the GFP affinity column, detergents, salts and ligand were added to their final concentrations, 1% (w/v) MNG-3/0.1% (w/v) CHS solution, 200 mM NaCl and 16 μM NT1, respectively. The mixture was homogenized using a hand-held glass homogenizer.

Apyrase (25 mU/mL, final concentration) and MgCl_2_ (2 mM, final concentration) were added to both samples, and solubilization was allowed to proceed for 1.5 h. Following ultra-centrifugation to remove insoluble membrane debris, the supernatants were mixed with the respective beads (0.2 mL) and incubated on an end-over-end roller overnight in the cold room. The beads from both mixtures were collected into two separate chromatography columns (Poly-Prep, Bio-Rad). Both columns were washed with wash buffer I (50 mM Tris pH 7.4, 30% (v/v) glycerol, 200 mM NaCl, 1 μM NT1, 100 μM tris(2-carboxyethyl)phosphine hydrochloride (TCEP), 0.1% (w/v) MNG-3/0.01% (w/v) CHS) and wash buffer II (50 mM Tris pH 7.4, 30% (v/v) glycerol, 200 mM NaCl, 1 μM NT1, 100 μM TCEP, 0.05% (w/v) MNG-3/0.005% (w/v) CHS). For elution, the resins of both columns were resuspended in 0.2 mL of wash buffer II, and 80 μg of purified 3C protease (in a volume of 50 μl) was added to both the columns. The mixture was incubated for 2 h at 4°C on an end-over-end roller. The protein was eluted in a total volume of 0.6 mL from each column. For removing 3C protease and the eYFP moiety, 20 mM imidazole and 50 μl of washed Ni^2+^-NTA resin was added to each elution, and the mixture was incubated for 2 h on an end-over-end roller. The flow-through (FT) was collected and beads were additionally washed with 0.2 mL of wash buffer II. The buffer was exchanged, using a PD minitrap G25 column (GE Healthcare), to the size-exclusion (SEC) buffer (50 mM Tris-HCl pH 7.4, 200 mM NaCl, 1 μM NT1, 100 μM TCEP, 0.05% (w/v) MNG-3/0.005% (w/v) CHS). The samples were separately concentrated to a volume of 100 μl and then used for analytical SEC. The analytical SEC was performed on a pre-equilibrated Superdex 200 Increase 5/150 GL column (GE Healthcare) installed on an ÄKTA-micro system. Fractions of 100 μl were collected, and 10 μl from the highest protein-containing fraction was analyzed by SDS-PAGE followed by silver staining (SilverQuest, ThermoFisher).

## Supporting information

S1 Fig Western blot of the subunits of the rNTR1*-Gα_i1/q_β_1_γ_1_ complex produced in insect cells. Representative western blot analysis to confirm the expression of all subunits in the transfected insect cells. Lane1, molecular weight standard. A cell lysate corresponding to about 10,000 cells (lane 2) was loaded and subjected to western blot analysis using anti-Gα_i1_ (detecting the receptor-Gα fusion), anti-RGSHis (detecting Gβ_1_) and anti-HA (detecting Gγ_1_) as primary antibodies and goat AF680 anti-rabbit (ThermoFisher) and donkey IRDye800 anti-mouse (Rockland) as secondary antibodies. Imaging was carried out using an infrared imaging system (LI-COR Odyssey Imaging System). All the subunits were found to migrate close to their theoretical molecular weight, (~76 kDa, receptor-Gα fusion; ~40 kDa, RGS(His)_10_-3C-Gβ_1_; ~9 kDa, HA-Gγ_1_). A very small fraction of possible dimer species of the receptor-Gα fusion, migrating just above 130 kDa, was also visible (indicated by an arrow). * rNTR1 mutant used: HTGH4-ΔICL3(B).
(TIF)

S2 Fig SDS-PAGE analysis of a typical purification of rNTR1*-Gα_i1/q_β_1_γ_1_ complex from insect cells using the NT-affinity resin. Membranes containing rNTR1*-Gα_i1/q_β_1_γ_1_ complex were solubilized in DM and the soluble portion was subjected to NT ligand-affinity chromatography, where the detergent was exchanged into OG. Lanes: (M) molecular weight marker; (1) flow-through of NT ligand-affinity column; (2) first wash of NT ligand-affinity column with OG-containing buffer; (3) second wash of NT ligand-affinity column with OG-containing buffer; (4) resin of NT ligand-affinity column after wash; (5) resin of NT ligand-affinity column after elution with 3C protease; (6) eluate of NT ligand-affinity column (1:3 dilution); (7) flow-through of Ni^2+^-NTA column (1:3 dilution). Note that in lane 5, a portion of the protein remained bound to the resin after elution. The problem may be circumvented by adding more 3C protease or with a longer incubation time prior to elution. *rNTR1 mutant used: HTGH4-ΔICL3(B). Abbreviations: DM, n-decyl-β-D-maltoside; OG, n-octyl-β-D-glucoside.
(TIF)

S3 Fig Size-exclusion chromatography elution profile of the purified rNTR1*-Gα_i1/q_β_1_γ_1_ complex in various detergents. Compilation of SEC elution profiles in various detergents. The complex was generated using the evolved NTR1 mutant HTGH4-ΔICL3(B). All chromatograms shown represent purifications of the fusion-complex carried out using the NT ligand-affinity purification strategy. The exchange to the detergent of choice was performed on the NT ligand-affinity column and the detergent of choice was then used in all the subsequent buffers. The small peaks at about 8 mL in DDM:CHS (**i)** and MNG:CHS (**ii**) indicate aggregated protein that may have been generated during the protein concentration step prior to loading onto the size-exclusion column. In DM (**iii**) and NG (**iv**) the protein remained highly monodisperse. In OG (**v**) there was a slight tendency for dimerization (small peak at about 11 ml). For exchange into NG or OG detergents, membrane solubilization was carried out in DM. Attempts of detergent exchange directly from DDM:CHS to NG or OG led to a significant loss of protein. The protein was not stable in HG detergent (data not shown). All the analytical gel filtrations were performed on a Superdex 200 Increase 10/300 GL column (GE Healthcare). All shown percentages indicate w/v of the detergent solution used. *rNTR1 mutant used: HTGH4-ΔICL3(B).
Abbreviations: DDM, n-dodecyl-β-D-maltoside; DM, n-decyl-β-D-maltoside; NG, n-nonyl-β-D-glucopyranoside; OG, n-octyl-β-D-glucoside; MNG-3, lauryl-maltose neopentyl glycol; CHS, cholesteryl hemisuccinate; HG, n-heptyl-β-D-glucopyranoside.
(TIF)

S4 Fig Plasmid map for pFL_m_rNTR1*_G-alpha i1/q MRGS His10 beta1 –HA Gamma 1. Representative plasmid map of the final vector obtained after Cre-Lox recombination of pFL_m_rNTR1*_G-alpha i1/q and pIDC MRGS His10 beta1- HA Gamma1 (pIDCβγ).
Abbreviations: Chloramphenicol (R), Chloramphenicol resistance gene; Gentamycin (R), gentamycin resistance gene; Ampicillin (R), ampicillin resistance gene; ColE1, high-copy number ColE1 origin of replication; R6K gamma origin, gamma origin of the plasmid R6K; pPH, polyhedrin promoter; Pp10, p10 promoter; LoxP, locus of cross-over in P1; Tn7R, right end of the Tn7 transposon; Tn7L, left end of the Tn7 transposon; SV-40-pA, polyadenylation signal (from simian virus 40); HSV TK pA, herpes simplex virus (HSV) thymidine kinase (TK) polyadenylation signal sequence; LIC site, ligation-independent cloning site; Melittin signal sequence, (MKFLVNVALVFMVVYISYIYA); rNTR1*G50-P389 ΔE273-T290 ΔIC3(B), rat neurotensin receptor mutant (with four residues, GPGS prior to residue G50 of the receptor, containing ΔICL3(B) deletion and C-terminally truncated at residue P389); G-alpha_i1/q_, chimeric Gα_i1/q_ (as described in the text); MRGS His 10, RGS decahistidine tag; 3C Protease, human rhinovirus (HRV) 3C protease cleavage site (LEVLFQGP); beta 1, human Gβ_1_ (as described in the text); HA-Gamma1, N-terminally hemagglutinin (YPYDVPDYA)-tagged human γ_1_ (as described in the text)
(TIF)

S1 Table Concentrations of tested detergents. Concentrations of all the tested detergents used in the respective buffers. All values indicate w/v in percentage. Abbreviations: DDM, n-dodecyl-β-D-maltoside; DM, n-decyl-β-D-maltoside; NG, n-nonyl-β-D-glucopyranoside; OG, n-octyl-β-D-glucoside; MNG-3, lauryl-maltose neopentyl glycol; CHS, cholesteryl hemisuccinate.
(DOCX)

## References

[1] Mahoney JP, Sunahara RK. Mechanistic insights into GPCR-G protein interactions. Curr Opin Struct Biol. 2016; 41: 247–254. 10.1016/j.sbi.2016.11.005 27871057 PMC5363957

[2] Chen Q, Iverson TM, Gurevich VV. Structural basis of arrestin-dependent signal transduction. Trends Biochem Sci. 2018; 43: 412–423. 10.1016/j.tibs.2018.03.005 29636212 PMC5959776

[3] Koehl A, Hu H, Maeda S, Zhang Y, Qu Q, Paggi JM, et al Structure of the micro-opioid receptor-Gi protein complex. Nature. 2018 10.1038/s41586-018-0219-7 29899455 PMC6317904

[4] Kang Y, Kuybeda O, de Waal PW, Mukherjee S, Van Eps N, Dutka P, et al Cryo-EM structure of human rhodopsin bound to an inhibitory G protein. Nature. 2018 10.1038/s41586-018-0215-y 29899450 PMC8054211

[5] Zhang Y, Sun B, Feng D, Hu H, Chu M, Qu Q, et al Cryo-EM structure of the activated GLP-1 receptor in complex with a G protein. Nature. 2017; 546: 248–253. 10.1038/nature22394 28538729 PMC5587415

[6] Griebel G. Neuropeptide receptor ligands for the treatment of schizophrenia: focus on neurotensin and tachykinins. Curr Pharm Des. 2015; 21: 3807–3812. 26044977 10.2174/1381612821666150605105859

[7] Souaze F, Dupouy S, Viardot-Foucault V, Bruyneel E, Attoub S, Gespach C, et al Expression of neurotensin and NT1 receptor in human breast cancer: a potential role in tumor progression. Cancer Res. 2006; 66: 6243–6249. 10.1158/0008-5472.CAN-06-0450 16778199

[8] Alifano M, Souaze F, Dupouy S, Camilleri-Broet S, Younes M, Ahmed-Zaid SM, et al Neurotensin receptor 1 determines the outcome of non-small cell lung cancer. Clin Cancer Res. 2010; 16: 4401–4410. 10.1158/1078-0432.CCR-10-0659 20810387

[9] Dupouy S, Viardot-Foucault V, Alifano M, Souaze F, Plu-Bureau G, Chaouat M, et al The neurotensin receptor-1 pathway contributes to human ductal breast cancer progression. PLoS One. 2009; 4: e4223 10.1371/journal.pone.0004223 19156213 PMC2626627

[10] Li J, Song J, Zaytseva YY, Liu Y, Rychahou P, Jiang K, et al An obligatory role for neurotensin in high-fat-diet-induced obesity. Nature. 2016; 533: 411–415. 10.1038/nature17662 27193687 PMC5484414

[11] St-Gelais F, Jomphe C, Trudeau LE. The role of neurotensin in central nervous system pathophysiology: what is the evidence? J Psychiatry Neurosci. 2006; 31: 229–245. 16862241 PMC1488904

[12] Zhao D, Pothoulakis C. Effects of NT on gastrointestinal motility and secretion, and role in intestinal inflammation. Peptides. 2006; 27: 2434–2444. 10.1016/j.peptides.2005.12.016 16872719

[13] Sarkar CA, Dodevski I, Kenig M, Dudli S, Mohr A, Hermans E, et al Directed evolution of a G protein-coupled receptor for expression, stability, and binding selectivity. Proc Natl Acad Sci U S A. 2008; 105: 14808–14813. 10.1073/pnas.0803103105 18812512 PMC2567449

[14] Schlinkmann KM, Hillenbrand M, Rittner A, Kunz M, Strohner R, Plückthun A. Maximizing detergent stability and functional expression of a GPCR by exhaustive recombination and evolution. J Mol Biol. 2012; 422: 414–428. 10.1016/j.jmb.2012.05.039 22683350

[15] Schlinkmann KM, Honegger A, Tureci E, Robison KE, Lipovsek D, Plückthun A. Critical features for biosynthesis, stability, and functionality of a G protein-coupled receptor uncovered by all-versus-all mutations. Proc Natl Acad Sci U S A. 2012; 109: 9810–9815. 10.1073/pnas.1202107109 22665811 PMC3382542

[16] Scott DJ, Kummer L, Egloff P, Bathgate RA, Plückthun A. Improving the apo-state detergent stability of NTS1 with CHESS for pharmacological and structural studies. Biochim Biophys Acta. 2014; 1838: 2817–2824. 10.1016/j.bbamem.2014.07.015 25064156

[17] Egloff P, Hillenbrand M, Klenk C, Batyuk A, Heine P, Balada S, et al Structure of signaling-competent neurotensin receptor 1 obtained by directed evolution in Escherichia coli. Proc Natl Acad Sci U S A. 2014; 111: E655–662. 10.1073/pnas.1317903111 24453215 PMC3926081

[18] Hillenbrand M, Schori C, Schöppe J, Plückthun A. Comprehensive analysis of heterotrimeric G-protein complex diversity and their interactions with GPCRs in solution. Proc Natl Acad Sci U S A. 2015; 112: E1181–1190. 10.1073/pnas.1417573112 25733868 PMC4371982

[19] Milligan G, Parenty G, Stoddart LA, Lane JR. Novel pharmacological applications of G-protein-coupled receptor-G protein fusions. Curr Opin Pharmacol. 2007; 7: 521–526. 10.1016/j.coph.2007.06.007 17689289

[20] Hildebrandt JD. Bring your own G protein. Mol Pharmacol. 2006; 69: 1079–1082. 10.1124/mol.106.022921 16436587

[21] Grisshammer R, Hermans E. Functional coupling with Galpha(q) and Galpha(i1) protein subunits promotes high-affinity agonist binding to the neurotensin receptor NTS-1 expressed in Escherichia coli. FEBS Lett. 2001; 493: 101–105. 11287004 10.1016/s0014-5793(01)02281-5

[22] Wenzel-Seifert K, Lee TW, Seifert R, Kobilka BK. Restricting mobility of Gsalpha relative to the beta2-adrenoceptor enhances adenylate cyclase activity by reducing Gsalpha GTPase activity. Biochem J. 1998; 334: 519–524. 9729456 10.1042/bj3340519 PMC1219717

[23] Marino SF. High-level production and characterization of a G-protein coupled receptor signaling complex. FEBS J. 2009; 276: 4515–4528. 10.1111/j.1742-4658.2009.07158.x 19645726

[24] Kang Y, Zhou XE, Gao X, He Y, Liu W, Ishchenko A, et al Crystal structure of rhodopsin bound to arrestin by femtosecond X-ray laser. Nature. 2015; 523: 561–567. 10.1038/nature14656 26200343 PMC4521999

[25] Bieniossek C, Richmond TJ, Berger I. MultiBac: multigene baculovirus-based eukaryotic protein complex production. Curr Protoc Protein Sci. 2008; Chapter 5: Unit 5 20. 10.1002/0471140864.ps0520s51 18429060

[26] Egloff P, Deluigi M, Heine P, Balada S, Plückthun A. A cleavable ligand column for the rapid isolation of large quantities of homogeneous and functional neurotensin receptor 1 variants from E. coli. Protein Expr Purif. 2015; 108: 106–114. 10.1016/j.pep.2014.10.006 25461958

[27] Hansen S, Stüber JC, Ernst P, Koch A, Bojar D, Batyuk A, et al Design and applications of a clamp for Green Fluorescent Protein with picomolar affinity. Sci Rep. 2017; 7: 16292 10.1038/s41598-017-15711-z 29176615 PMC5701241

[28] Milligan G. Principles: extending the utility of [35S]GTP gamma S binding assays. Trends Pharmacol Sci. 2003; 24: 87–90. 12559773 10.1016/s0165-6147(02)00027-5

[29] Tucker J, Grisshammer R. Purification of a rat neurotensin receptor expressed in Escherichia coli. Biochem J. 1996; 317 (Pt 3): 891–899. 8760379 10.1042/bj3170891 PMC1217569

[30] Zhou Q, Zhou N, Wang HW. Particle segmentation algorithm for flexible single particle reconstruction. Biophys Rep. 2017; 3: 43–55. 10.1007/s41048-017-0038-7 28782000 PMC5515998

[31] Kleinau G, Jaeschke H, Worth CL, Mueller S, Gonzalez J, Paschke R, et al Principles and determinants of G-protein coupling by the rhodopsin-like thyrotropin receptor. PLoS One. 2010; 5: e9745 10.1371/journal.pone.0009745 20305779 PMC2841179

[32] Sato T, Kawasaki T, Mine S, Matsumura H. Functional role of the C-terminal amphipathic helix 8 of olfactory receptors and other G protein-coupled receptors. Int J Mol Sci. 2016; 17 10.3390/ijms17111930 27869740 PMC5133925

[33] Liang YL, Khoshouei M, Radjainia M, Zhang Y, Glukhova A, Tarrasch J, et al Phase-plate cryo-EM structure of a class B GPCR-G-protein complex. Nature. 2017; 546: 118–123. 10.1038/nature22327 28437792 PMC5832441

[34] Feierler J, Wirth M, Welte B, Schüssler S, Jochum M, Faussner A. Helix 8 plays a crucial role in bradykinin B(2) receptor trafficking and signaling. J Biol Chem. 2011; 286: 43282–43293. 10.1074/jbc.M111.256909 22016392 PMC3234833

[35] Heakal Y, Woll MP, Fox T, Seaton K, Levenson R, Kester M. Neurotensin receptor-1 inducible palmitoylation is required for efficient receptor-mediated mitogenic-signaling within structured membrane microdomains. Cancer Biol Ther. 2011; 12: 427–435. 10.4161/cbt.12.5.15984 21725197 PMC3219081

[36] Holst B, Hastrup H, Raffetseder U, Martini L, Schwartz TW. Two active molecular phenotypes of the tachykinin NK1 receptor revealed by G-protein fusions and mutagenesis. J Biol Chem. 2001; 276: 19793–19799. 10.1074/jbc.M100621200 11279104

[37] Fitzgerald DJ, Schaffitzel C, Berger P, Wellinger R, Bieniossek C, Richmond TJ, et al Multiprotein expression strategy for structural biology of eukaryotic complexes. Structure. 2007; 15: 275–279. 10.1016/j.str.2007.01.016 17355863

[38] Trowitzsch S, Bieniossek C, Nie Y, Garzoni F, Berger I. New baculovirus expression tools for recombinant protein complex production. J Struct Biol. 2010; 172: 45–54. 10.1016/j.jsb.2010.02.010 20178849

[39] Rasmussen SG, DeVree BT, Zou Y, Kruse AC, Chung KY, Kobilka TS, et al Crystal structure of the beta2 adrenergic receptor-Gs protein complex. Nature. 2011; 477: 549–555. 10.1038/nature10361 21772288 PMC3184188

[40] Milic D, Veprintsev DB. Large-scale production and protein engineering of G protein-coupled receptors for structural studies. Front Pharmacol. 2015; 6: 66 10.3389/fphar.2015.00066 25873898 PMC4379943

[41] Maeda S, Sun D, Singhal A, Foggetta M, Schmid G, Standfuss J, et al Crystallization scale preparation of a stable GPCR signaling complex between constitutively active rhodopsin and G-protein. PLoS One. 2014; 9: e98714 10.1371/journal.pone.0098714 24979345 PMC4076187

[42] Hassell AM, An G, Bledsoe RK, Bynum JM, Carter HL 3rd, Deng SJ, et al Crystallization of protein-ligand complexes. Acta Crystallogr D Biol Crystallogr. 2007; 63: 72–79. 10.1107/S0907444906047020 17164529 PMC2483499

[43] Schmitz AL, Schrage R, Gaffal E, Charpentier TH, Wiest J, Hiltensperger G, et al A cell-permeable inhibitor to trap Galphaq proteins in the empty pocket conformation. Chem Biol. 2014; 21: 890–902. 10.1016/j.chembiol.2014.06.003 25036778 PMC4337399

[44] Zhang X, Stevens RC, Xu F. The importance of ligands for G protein-coupled receptor stability. Trends Biochem Sci. 2015; 40: 79–87. 10.1016/j.tibs.2014.12.005 25601764

[45] Krumm BE, Lee S, Bhattacharya S, Botos I, White CF, Du H, et al Structure and dynamics of a constitutively active neurotensin receptor. Sci Rep. 2016; 6: 38564 10.1038/srep38564 27924846 PMC5141500

[46] Flock T, Ravarani CNJ, Sun D, Venkatakrishnan AJ, Kayikci M, Tate CG, et al Universal allosteric mechanism for Galpha activation by GPCRs. Nature. 2015; 524: 173–179. 10.1038/nature14663 26147082 PMC4866443

[47] Gay P, Le Coq D, Steinmetz M, Berkelman T, Kado CI. Positive selection procedure for entrapment of insertion sequence elements in gram-negative bacteria. J Bacteriol. 1985; 164: 918–921. 2997137 10.1128/jb.164.2.918-921.1985 PMC214340

[48] Witholt B, Boekhout M, Brock M, Kingma J, Heerikhuizen HV, Leij LD. An efficient and reproducible procedure for the formation of spheroplasts from variously grown Escherichia coli. Anal Biochem. 1976; 74: 160–170. 786067 10.1016/0003-2697(76)90320-1

[49] Bieniossek C, Imasaki T, Takagi Y, Berger I. MultiBac: expanding the research toolbox for multiprotein complexes. Trends Biochem Sci. 2012; 37: 49–57. 10.1016/j.tibs.2011.10.005 22154230 PMC7127121

[50] Haldimann A, Prahalad MK, Fisher SL, Kim SK, Walsh CT, Wanner BL. Altered recognition mutants of the response regulator PhoB: a new genetic strategy for studying protein-protein interactions. Proc Natl Acad Sci U S A. 1996; 93: 14361–14366. 8962056 10.1073/pnas.93.25.14361 PMC26137

[51] Fitzgerald DJ, Berger P, Schaffitzel C, Yamada K, Richmond TJ, Berger I. Protein complex expression by using multigene baculoviral vectors. Nat Methods. 2006; 3: 1021–1032. 10.1038/nmeth983 17117155

[52] Wasilko DJ, Lee SE, Stutzman-Engwall KJ, Reitz BA, Emmons TL, Mathis KJ, et al The titerless infected-cells preservation and scale-up (TIPS) method for large-scale production of NO-sensitive human soluble guanylate cyclase (sGC) from insect cells infected with recombinant baculovirus. Protein Expr Purif. 2009; 65: 122–132. 10.1016/j.pep.2009.01.002 19174191

