# Supplementary figures and images for: In vivo assembly and large-scale purification of a GPCR - Gα fusion with Gβγ, and characterization of the active complex
Source: PLoS One. 2019 Jan 8;14(1):e0210131. doi: 10.1371/journal.pone.0210131 (PMC6324789; doi:10.1371/journal.pone.0210131)

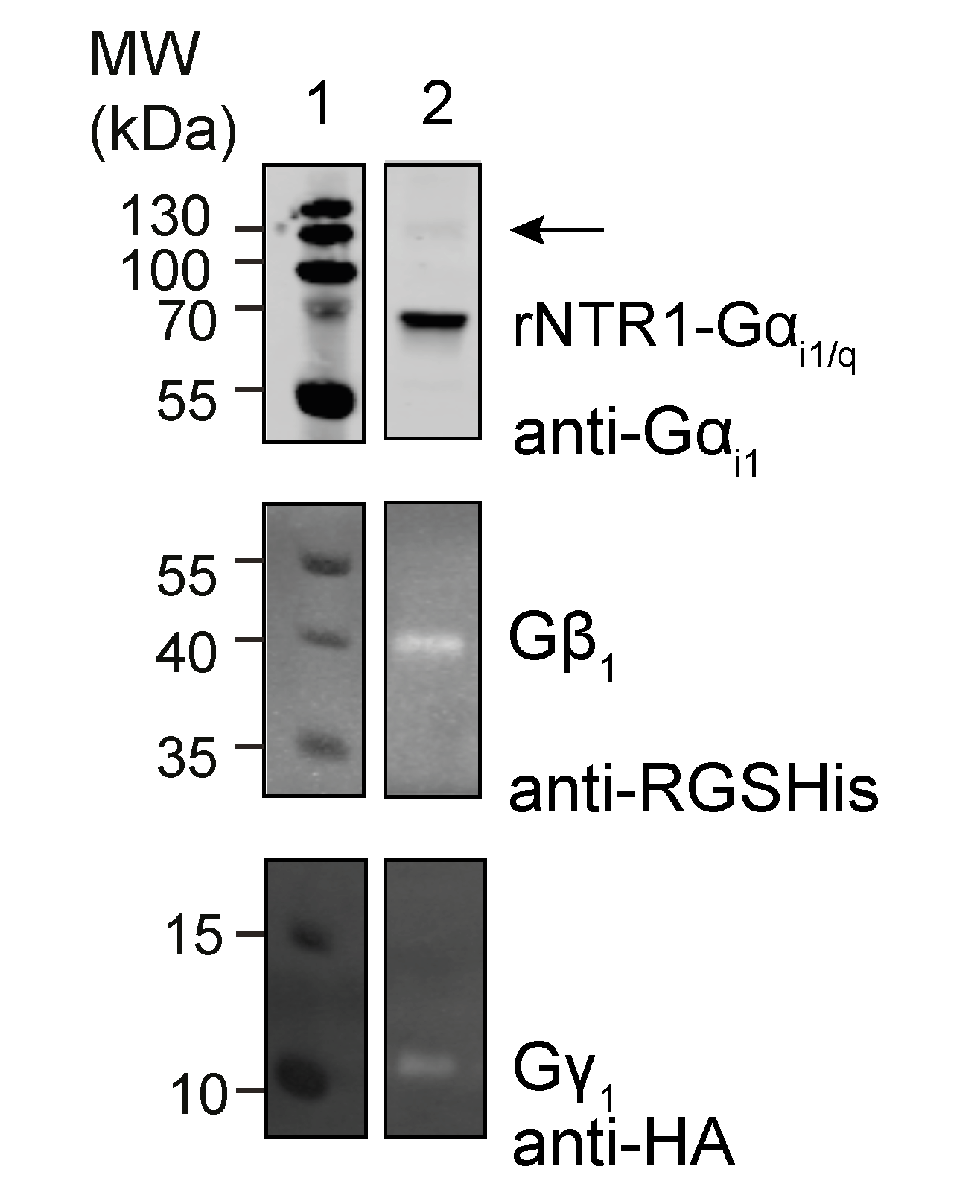

Supplement: S1 Fig — Representative western blot analysis to confirm the expression of all subunits in the transfected insect cells. Lane1, molecular weight standard. A cell lysate corresponding to about 10,000 cells (lane 2) was loaded and subjected to western blot analysis using anti-Gαi1 (detecting the receptor-Gα fusion), anti-RGSHis (detecting Gβ1) and anti-HA (detecting Gγ1) as primary antibodies and goat AF680 anti-rabbit (ThermoFisher) and donkey IRDye800 anti-mouse (Rockland) as secondary antibodies. Imaging was carried out using an infrared imaging system (LI-COR Odyssey Imaging System). All the subunits were found to migrate close to their theoretical molecular weight, (~76 kDa, receptor-Gα fusion; ~40 kDa, RGS(His)10-3C-Gβ1; ~9 kDa, HA-Gγ1). A very small fraction of possible dimer species of the receptor-Gα fusion, migrating just above 130 kDa, was also visible (indicated by an arrow). * rNTR1 mutant used: HTGH4-ΔICL3(B). (TIF) [file pone.0210131.s001.tif]

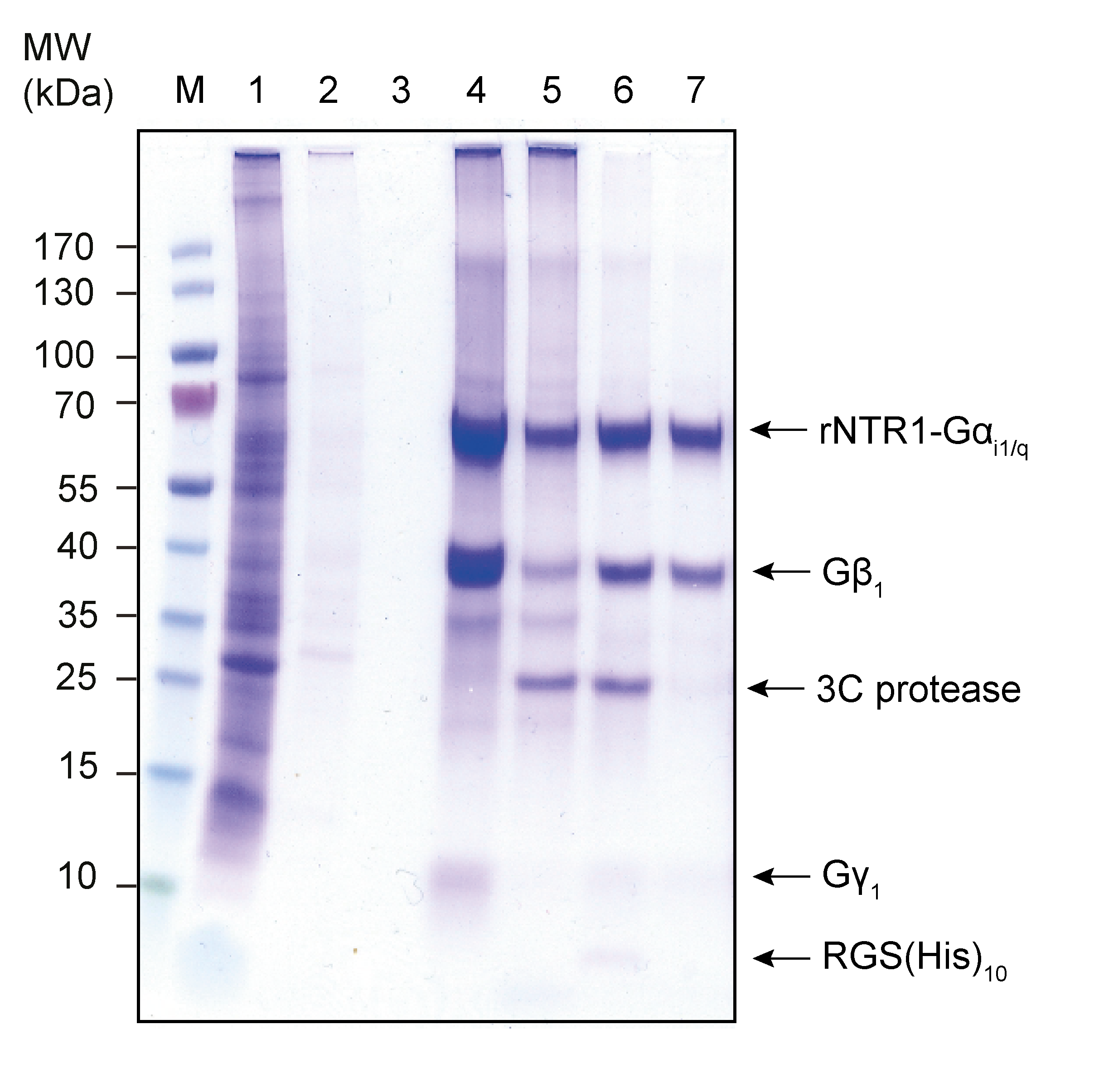

Supplement: S2 Fig — Membranes containing rNTR1*-Gαi1/qβ1γ1 complex were solubilized in DM and the soluble portion was subjected to NT ligand-affinity chromatography, where the detergent was exchanged into OG. Lanes: (M) molecular weight marker; (1) flow-through of NT ligand-affinity column; (2) first wash of NT ligand-affinity column with OG-containing buffer; (3) second wash of NT ligand-affinity column with OG-containing buffer; (4) resin of NT ligand-affinity column after wash; (5) resin of NT ligand-affinity column after elution with 3C protease; (6) eluate of NT ligand-affinity column (1:3 dilution); (7) flow-through of Ni2+-NTA column (1:3 dilution). Note that in lane 5, a portion of the protein remained bound to the resin after elution. The problem may be circumvented by adding more 3C protease or with a longer incubation time prior to elution. *rNTR1 mutant used: HTGH4-ΔICL3(B). Abbreviations: DM, n-decyl-β-D-maltoside; OG, n-octyl-β-D-glucoside. (TIF) [file pone.0210131.s002.tif]

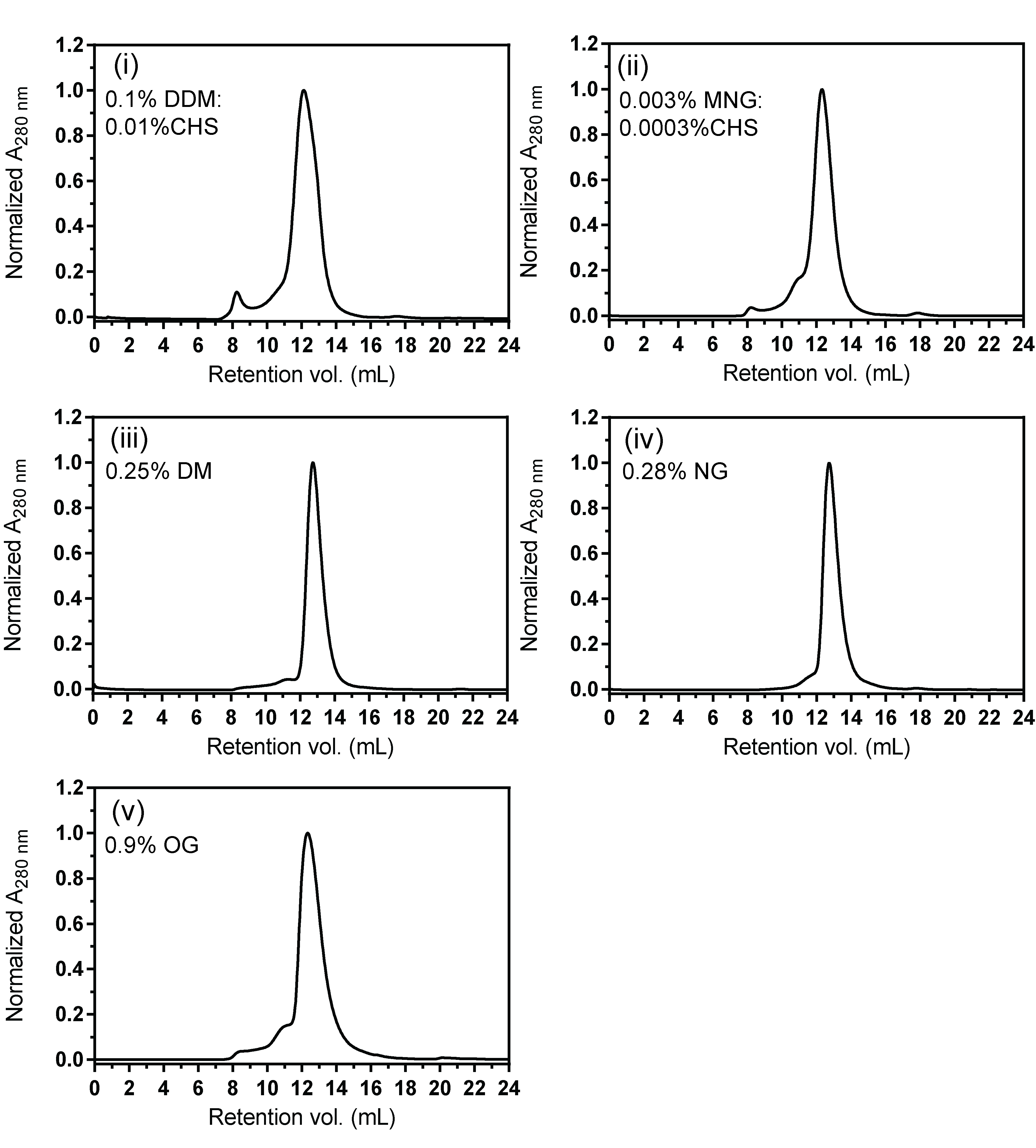

Supplement: S3 Fig — Compilation of SEC elution profiles in various detergents. The complex was generated using the evolved NTR1 mutant HTGH4-ΔICL3(B). All chromatograms shown represent purifications of the fusion-complex carried out using the NT ligand-affinity purification strategy. The exchange to the detergent of choice was performed on the NT ligand-affinity column and the detergent of choice was then used in all the subsequent buffers. The small peaks at about 8 mL in DDM:CHS (i) and MNG:CHS (ii) indicate aggregated protein that may have been generated during the protein concentration step prior to loading onto the size-exclusion column. In DM (iii) and NG (iv) the protein remained highly monodisperse. In OG (v) there was a slight tendency for dimerization (small peak at about 11 ml). For exchange into NG or OG detergents, membrane solubilization was carried out in DM. Attempts of detergent exchange directly from DDM:CHS to NG or OG led to a significant loss of protein. The protein was not stable in HG detergent (data not shown). All the analytical gel filtrations were performed on a Superdex 200 Increase 10/300 GL column (GE Healthcare). All shown percentages indicate w/v of the detergent solution used. *rNTR1 mutant used: HTGH4-ΔICL3(B). Abbreviations: DDM, n-dodecyl-β-D-maltoside; DM, n-decyl-β-D-maltoside; NG, n-nonyl-β-D-glucopyranoside; OG, n-octyl-β-D-glucoside; MNG-3, lauryl-maltose neopentyl glycol; CHS, cholesteryl hemisuccinate; HG, n-heptyl-β-D-glucopyranoside. (TIF) [file pone.0210131.s003.tif]

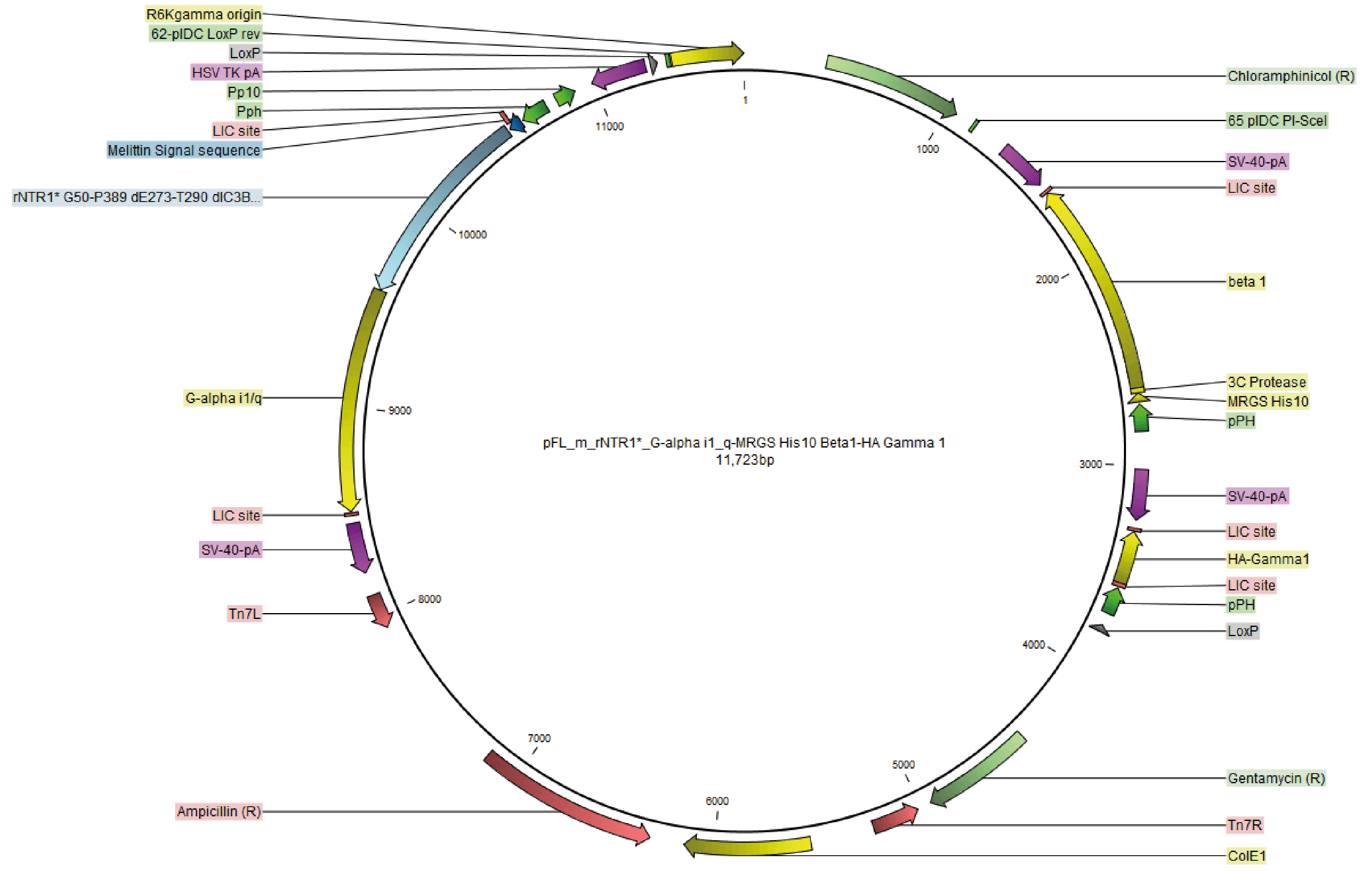

Supplement: S4 Fig — Representative plasmid map of the final vector obtained after Cre-Lox recombination of pFL_m_rNTR1*_G-alpha i1/q and pIDC MRGS His10 beta1- HA Gamma1 (pIDCβγ). Abbreviations: Chloramphenicol (R), Chloramphenicol resistance gene; Gentamycin (R), gentamycin resistance gene; Ampicillin (R), ampicillin resistance gene; ColE1, high-copy number ColE1 origin of replication; R6K gamma origin, gamma origin of the plasmid R6K; pPH, polyhedrin promoter; Pp10, p10 promoter; LoxP, locus of cross-over in P1; Tn7R, right end of the Tn7 transposon; Tn7L, left end of the Tn7 transposon; SV-40-pA, polyadenylation signal (from simian virus 40); HSV TK pA, herpes simplex virus (HSV) thymidine kinase (TK) polyadenylation signal sequence; LIC site, ligation-independent cloning site; Melittin signal sequence, (MKFLVNVALVFMVVYISYIYA); rNTR1*G50-P389 ΔE273-T290 ΔIC3(B), rat neurotensin receptor mutant (with four residues, GPGS prior to residue G50 of the receptor, containing ΔICL3(B) deletion and C-terminally truncated at residue P389); G-alphai1/q, chimeric Gαi1/q (as described in the text); MRGS His 10, RGS decahistidine tag; 3C Protease, human rhinovirus (HRV) 3C protease cleavage site (LEVLFQGP); beta 1, human Gβ1 (as described in the text); HA-Gamma1, N-terminally hemagglutinin (YPYDVPDYA)-tagged human γ1 (as described in the text) (TIF) [file pone.0210131.s004.tif]
