# Supplementary material for: In vivo assembly and large-scale purification of a GPCR - Gα fusion with Gβγ, and characterization of the active complex
Source: PLoS One. 2019 Jan 8;14(1):e0210131. doi: 10.1371/journal.pone.0210131 (PMC6324789; doi:10.1371/journal.pone.0210131)
Supplement: S1 Table — Concentrations of all the tested detergents used in the respective buffers. All values indicate w/v in percentage. Abbreviations: DDM, n-dodecyl-β-D-maltoside; DM, n-decyl-β-D-maltoside; NG, n-nonyl-β-D-glucopyranoside; OG, n-octyl-β-D-glucoside; MNG-3, lauryl-maltose neopentyl glycol; CHS, cholesteryl hemisuccinate. (DOCX) [file pone.0210131.s005.docx]

| **S1 Table: Concentrations of tested detergents** | | | | |  | |
| --- | --- | --- | --- | --- | --- | --- |
|  | DDM/CHS (w/v %) | MNG-3/CHS (w/v %) | DM  (w/v %) | NG  (w/v %) | | OG  (w/v %) |
| Solubilization | 1/0.1 | 1/0.1 | 1.50 | - | | - |
| Wash buffer-1 | 0.5/0.05 | 0.1/0.01 | 0.40 | 0.50 | | 1.50 |
| Wash buffer-2 | 0.1/0.01 | 0.05/0.005 | 0.25 | 0.30 | | 1.00 |
| Reverse Ni^2+^ | 0.1/0.01 | 0.05/0.005 | 0.25 | 0.30 | | 1.00 |
| Size-exclusion buffer | 0.1/0.01 | 0.003/0.0003 | 0.25 | 0.28 | | 0.90 |

Concentrations of all the tested detergents used in the respective buffers. All values indicate w/v in percentage. Abbreviations: DDM, n-dodecyl-β-D-maltoside; DM, n-decyl-β-D-maltoside; NG, n-nonyl-β-D-glucopyranoside; OG, n-octyl-β-D-glucoside; MNG-3, lauryl-maltose neopentyl glycol; CHS, cholesteryl hemisuccinate.
